# Exosomal miR-17-5p derived from epithelial cells is involved in aberrant epithelium-fibroblast crosstalk and induces the development of oral submucosal fibrosis

**DOI:** 10.1038/s41368-024-00302-2

**Published:** 2024-06-20

**Authors:** Changqing Xie, Liang Zhong, Hui Feng, Rifu Wang, Yuxin Shi, Yonglin Lv, Yanjia Hu, Jing Li, Desheng Xiao, Shuang Liu, Qianming Chen, Yongguang Tao

**Affiliations:** 1 NHC Key Laboratory of Carcinogenesis, Cancer Research Institute, School of Basic Medicine Sciences, Central South University, Changsha, China; 2 Hospital of Stomatology and Key Laboratory of Oral Biomedical Research of Zhejiang Province, School of Stomatology, Zhejiang University School of Medicine, Hangzhou, China; 3 Hunan Key Laboratory of Oral Health Research & Hunan 3D Printing Engineering Research Center of Oral Care & Hunan Clinical Research Center of Oral Major Diseases and Oral Health & Xiangya Stomatological Hospital & Xiangya School of Stomatology, Central South University, Changsha, China; 4 State Key Laboratory of Oral Diseases & National Center for Stomatology & National Clinical Research Center for Oral Diseases & Chinese Academy of Medical Sciences Research Unit of Oral Carcinogenesis and Management, West China Hospital of Stomatology, Sichuan University, Chengdu, China; 5 Department of Oncology, Institute of Medical Sciences, National Clinical Research Center for Geriatric Disorders, Xiangya Hospital, Central South University, Changsha, China

**Keywords:** Mechanisms of disease, Oral diseases

## Abstract

Oral submucous fibrosis (OSF) is a chronic and inflammatory mucosal disease caused by betel quid chewing, which belongs to oral potentially malignant disorders. Abnormal fibroblast differentiation leading to disordered collagen metabolism is the core process underlying OSF development. The epithelium, which is the first line of defense against the external environment, can convert external signals into pathological signals and participate in the remodeling of the fibrotic microenvironment. However, the specific mechanisms by which the epithelium drives fibroblast differentiation remain unclear. In this study, we found that Arecoline-exposed epithelium communicated with the fibrotic microenvironment by secreting exosomes. MiR-17-5p was encapsulated in epithelial cell-derived exosomes and absorbed by fibroblasts, where it promoted cell secretion, contraction, migration and fibrogenic marker (α-SMA and collagen type I) expression. The underlying molecular mechanism involved miR-17-5p targeting Smad7 and suppressing the degradation of TGF-β receptor 1 (TGFBR1) through the E3 ubiquitination ligase WWP1, thus facilitating downstream TGF-β pathway signaling. Treatment of fibroblasts with an inhibitor of miR-17-5p reversed the contraction and migration phenotypes induced by epithelial-derived exosomes. Exosomal miR-17-5p was confirmed to function as a key regulator of the phenotypic transformation of fibroblasts. In conclusion, we demonstrated that Arecoline triggers aberrant epithelium-fibroblast crosstalk and identified that epithelial cell-derived miR-17-5p mediates fibroblast differentiation through the classical TGF-β fibrotic pathway, which provided a new perspective and strategy for the diagnosis and treatment of OSF.

## Introduction

Oral submucous fibrosis (OSF) is a connective tissue disease characterized by disordered epithelial structure, inflammation, vascular abnormalities and extracellular matrix accumulation.^1–3^ Numerous studies have indicated that chewing betel quid-related products is the main etiology of OSF.^4–6^ There was a high degree of overlap between OSF patients and betel quid chewing patients, mainly in Southeast Asian countries. Alarmingly, as an oral potential malignant disorder, approximately 1.5% to 10% of patients with OSF are at risk of malignant transformation.^7,8^ Although great progress has been made in determining the molecular mechanisms underlying disease pathogenesis in recent decades, no specific disease-modifying treatments are currently available.

Fibroblasts are a heterogeneous group of cells with multidifferentiation potential that maintain tissue homeostasis.^9,10^ Under pathological conditions, resting fibroblasts can differentiate into myofibroblasts with an anabolic phenotype due to continuous induction and chemotaxis by inflammatory factors.^11,12^ Myofibroblasts exhibit characteristics of both smooth muscle cells (high expression of α-SMA and collagen type I) and fibroblasts, which endows these cells with a highly contractile and secretory phenotype.^13–15^ Given their strong ability to remodel the extracellular matrix, myofibroblasts participate in oxidative stress, energy metabolism, autophagy, senescence and other pathological changes in the fibrotic microenvironment, providing an essential “hotbed” for the development of fibrosis.^16–18^ Therefore, revealing the key molecules that regulate myofibroblast differentiation and related signaling pathways is an important strategy for the prevention and treatment of OSF.

The epithelium is the physical and physiological barrier of the oral cavity.^19,20^ When exposed to injury, the subsequent cascade of inflammatory factors participates in shaping the fibrotic microenvironment.^21,22^ Exosomes, which are vesicles with a phospholipid bilayer, have been identified as novel modulators of cell-to-cell communication.^23^ Exosomes can deliver “cargo” such as liposomes, proteins, RNA and DNA, which absorbed by recipient cells to participate regulating the gene expression, differentiation and function of recipient cells.^24–26^ Importantly, exposure to stimuli can modify exosomes components to affect tissue remodeling via cell-to-cell communication.^27^ However, the cell types that are related to exosomes-mediated crosstalk and their paracrine effects on OSF development are unclear. There is increasing evidence that exosomal miRNAs can mediate phenotypic changes in fibroblasts, thereby participating in the progression of pulmonary fibrosis, liver fibrosis, kidney fibrosis, and skin fibrosis. In this study, we initially reported the role of epithelium-derived exosomal miRNAs in the development of OSF, and we explored the biological function of miR-17-5p in regulating fibroblast differentiation as well as the underlying mechanism.

In the present study, we hypothesized that exosomes derived from epithelial cells by exposure to Arecoline disrupt the epithelium-fibroblast crosstalk and promote myofibroblast differentiation. First, we demonstrated that Arecoline could induced the release of exosomes from epithelial cells, which could promote collagen contraction, secretion and migration after being absorbed by fibroblasts. Moreover, we revealed that miR-17-5p was highly expressed in exosomes from Arecoline-treated epithelial cells and OSF tissues. Mechanistically, it was demonstrated that miR-17-5p could target Smad7 and maintain TGF-β receptor 1 (TGFBR1) expression by inhibiting its E3 ubiquitination ligase WWP1. In summary, we evaluated the expression of miR-17-5p in exosomes derived from epithelial cells that were exposed to Arecoline and investigated the mechanism by which exosomal miR-17-5p contributes to fibrogenesis.

## Results

### Arecoline induces the release of exosomes from epithelial cells

Exosomes mediate cell‒cell communication through the transfer of their cargo and are involved in extracellular matrix homeostasis.^28^ Thus, we predicted that Arecoline (20 μg/mL, 48 h) can induce the release of exosomes from epithelial cells and affect the functional phenotype of fibroblasts. Exosomes were isolated from PBS- and arecoline-treated epithelial cell media via differential centrifugation, and then, these exosomes were characterized and quantified via TEM, NTA and Immunoblotting (Fig. S1a–c). TEM revealed nanovesicles that were round and had bilayered membranes; these findings are consistent with the characteristic shape and size range of exosomes. NTA revealed that the vesicles that were secreted by PBS- and Arecoline-treated epithelial cells were mainly 139 nm and 140 nm, respectively. Immunoblotting of lysates from purified nanovesicles revealed that exosomal markers, including CD9, CD63, CD81 (tetraspanins), TSG101 (ESCRT-I complex subunit), and Hsp70, were highly expressed in exosomes isolated from PBS- and Arecoline-treated epithelial cells. Calnexin (endoplasmic reticulum membrane) and GAPDH were barely expressed in the exosomes. Moreover, treatment of epithelial cells with GW4869, which is an inhibitor of exosome biosynthesis, inhibited Arecoline-induced exosome release from epithelial cells (Fig. S1d). These results indicated that the nanovesicles that were isolated from PBS- and Arecoline-treated epithelial cells presented characteristics that were typical of exosomes.

### Epithelial cell-derived exosomes are responsible for the myofibroblast differentiation phenotype

**Fig. 1:**
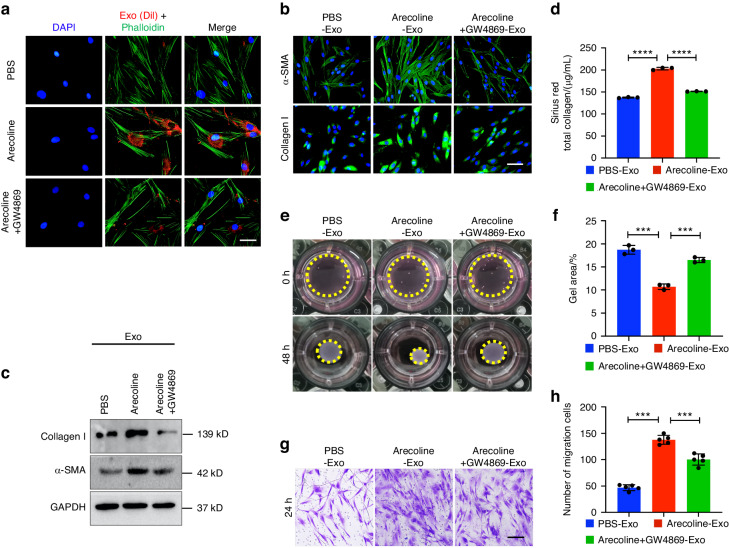
Epithelial cell-derived exosomes are transported to the primary fibroblasts, which promotes the myofibroblast differentiation phenotype. PBS-Exo (exosomes derived from epithelial cells treated with PBS), Arecoline-Exo (exosomes derived from epithelial cells treated with Arecoline), Arecoline+GW4869-Exo (exosomes derived from epithelial cells treated with Arecoline and GW4869). **a** Fibroblasts was incubated with DiI-labeled (red) exosomes from epithelial cells (50 μg) for 30 min and fixed for fluorescence staining. Nuclei were stained with DAPI (blue). Scale bars, 100 μm. **b** Immunostaining for fibrotic markers collagen type I and α-SMA in primary fibroblast with PBS-, Arecoline- and Arecoline+GW4869-Exo was evaluated by microscopy. DAPI (blue) was used for nuclear staining. Scale bar, 100 μm. **c** Immunoblotting of collagen type I and α-SMA by PBS-, Arecoline- and Arecoline+GW4869-Exo in primary fibroblasts. Sirius Red total collagen assay (**d**), collagen contraction assay (**e**, **f**) and transwell migration assay (**g**, **h**) were determined for fibroblast. *n* = 3–5 technical replicates, representative of two or three assays. Scale bars, 100 μm. Statistics: mean ± SEM, unpaired, one-way ANOVA (**d**, **f**, **h**), ***P < 0.001, ****P < 0.000 1

Therefore, we hypothesized that Arecoline-induced epithelial cell-derived exosomes might participate in fibroblast differentiation. To visualize exosomes transfer, we first incubated fibroblasts with epithelial cell-derived exosomes that had been labeled with the DiI stain for 24 h, and then, we evaluated exosomes uptake by measuring the intensity of the red signal in fibroblasts (Fig. 1a). The results showed robust exosomes signals in the cytoplasm of fibroblasts that were incubated with DiI-labeled exosomes, suggesting that the exosomes were successfully taken up by the fibroblasts. The levels of collagen type I and α-SMA were increased in fibroblasts that were incubated with exosomes derived from Arecoline-treated epithelial cells (Fig. 1b, c), and these expression levels increased in a dose-dependent manner with increasing exosomes concentration (Fig. S2a–c). Moreover, we verified that the protein content of collagen type I and α-SMA was equivalent in the exosomes derived from PBS-, Arecoline- and GW4869-treated epithelial cells, excluding the possibility of protein level regulation in exosomes (Fig. S2d). In addition, we collected supernatants of fibroblast cultures and found that the total collagen content was significantly increased in the group that was incubated with Arecoline-treated epithelial cell-derived exosomes (Fig. 1d). The migration (Fig. 1e, f) and contraction abilities of fibroblasts were also significantly increased by treatment with epithelial cell-derived exosomes (Fig. 1g, h). Consistent with these results, GW4869 inhibited collagen type I and α-SMA expression, collagen secretion, and contraction and migration of fibroblasts after treatment with epithelial cell-derived exosomes (Fig. 1a–h). These data suggested that exosomes secreted by Arecoline-treated epithelial cells could modulate the phenotypic transformation of fibroblasts.

### miR-17-5p is enriched in OSF epithelial cells-derived exosomes

**Fig. 2:**
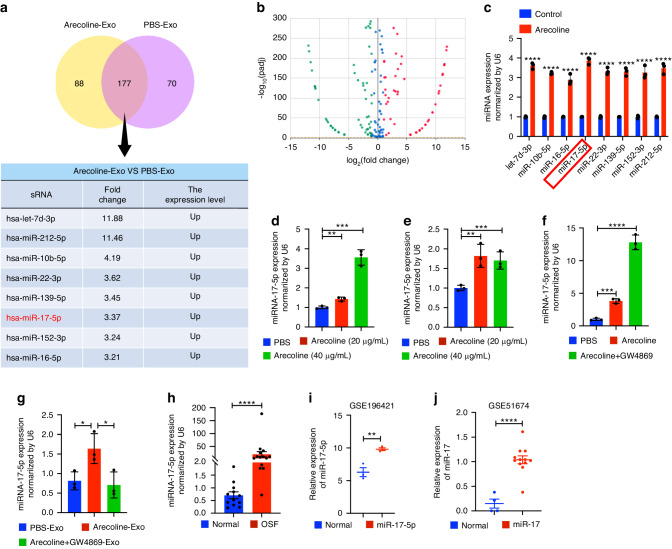
Arecoline induced the expression of miR-17-5p in epithelial cell exosomes. PBS-Exo (exosomes derived from epithelial cells treated with PBS), Arecoline-Exo (exosomes derived from epithelial cells treated with Arecoline), Arecoline+GW4869-Exo (exosomes derived from epithelial cells treated with Arecoline and GW4869), U6 was used as an internal control for qRT-PCR. The exosomes RNA–seq (**a**) and volcano plot (**b**) identified top 8 up-regulated miRNAs (change 1.5-fold) of 177 expressed differential genes in PBS-Exo and Arecoline-Exo. **c** qRT-PCR validation of exosomes miRNAs from PBS-Exo and Arecoline-Exo. The levels of miR-17-5p in epithelial cells (**d**) and exosomes (**e**) from its cell culture medium treated with PBS and Arecoline were determined by qRT-PCR. **f** The levels of miR-17-5p in epithelial cells treated with PBS, Arecoline and Arecoline+GW4869 were determined by qRT-PCR. **g** The levels of miR-17-5p in fibroblast after 48 h co-culturing with PBS-Exo, Arecoline-Exo and Arecoline+GW4869-Exo. **h** The levels of miR-17-5p in normal (*n* = 12) or OSF (*n* = 17) tissues. **i** miR-17-5p expression in normal (*n* = 3) and cardiac fibrosis patients (*n* = 3) from a public dataset (GSE196421). **j** miR-17 expression in normal (*n* = 4) and diabetic nephropathy kidney fibrosis patients (*n* = 12) from a public dataset (GSE51674). Statistics: mean ± SEM, unpaired, two-tailed Student’s t test (**c**, **h**, **i**, **j**) or one-way ANOVA (**d**–**g**), *P < 0.05, **P < 0.01, ***P < 0.001, ****P < 0.000 1

Next, we compared the differences in miRNA expression between exosomes derived from PBS- and Arecoline (20 μg/mL, 48 h)-treated epithelial cells by RNA-seq. We further analyzed the expression of the top 8 upregulated miRNAs in both groups (Fig. 2a, b). By qRT‒PCR, we found that miR-17-5p was the most differentially expressed miRNA in exosomes (Fig. 2c). Moreover, the expression of miR-17-5p was upregulated in a dose-dependent manner in arecoline-treated epithelial cells (Fig. 2d) and in exosomes in the corresponding supernatants (Fig. 2e). Interestingly, treatment with GW4869 resulted in the accumulation of miR-17-5p in epithelial cells (Fig. 2f). On the other hand, incubation with Arecoline-induced exosomes similarly upregulated miR-17-5p expression in fibroblasts, and this effect was blocked by GW4869 (Fig. 2g). To determine whether miR-17-5p is associated with the progression of fibrosis, miR-17-5p expression levels in OSF tissues were analyzed (Fig. 2h). We further analyzed miR-17-5p expression in tissues from patients with cardiac fibrosis and diabetic nephropathy kidney fibrosis using GEO datasets (Fig. 2i, j). On average, the expression of miR-17-5p was higher in fibrotic tissues than in normal tissues.

We then assessed the miR-17-5p levels in a mouse model of OSF. After 6 weeks of exposure to Bleomycin (Fig. S3a), the mice developed a connective tissue remodeling phenotype. Scanning electron microscopy (SEM) revealed that, compared with that in the PBS group, the structure of collagen fiber accumulation was apparent and collagen fiber arrangement was disordered in the Bleomycin group (Fig. S3b). HE, Masson’s trichrome and Sirius Red staining confirmed that collagen deposition and thickening as well as blood vessel formation were reduced in the lamina propria of the oral mucosa (Fig. S3c, d). A hydroxyproline experiment showed that the collagen content in the local tissues of the oral mucosa was significantly increased (Fig. S3e). Immunoblotting revealed that the protein levels of collagen type I, Fibronectin and α-SMA were increased in the mucosa of mice exposed to Bleomycin (Fig. S3k). These results indicated that Bleomycin successfully induced OSF in mice, which was consistent with previous research. qRT‒PCR analysis demonstrated that miR-17-5p was upregulated in blood-derived exosomes (Fig. S3l) and oral mucosal tissues (Fig. S3m) from Bleomycin-treated mice compared that in samples from control mice. Interestingly, these results were also confirmed in a mouse skin fibrosis model (Fig. S3f–o). These results indicated that the upregulation of miR-17-5p may be involved in the progression of fibrosis.

### Exosomal miR-17-5p is required for the myofibroblast differentiation phenotype

**Fig. 3:**
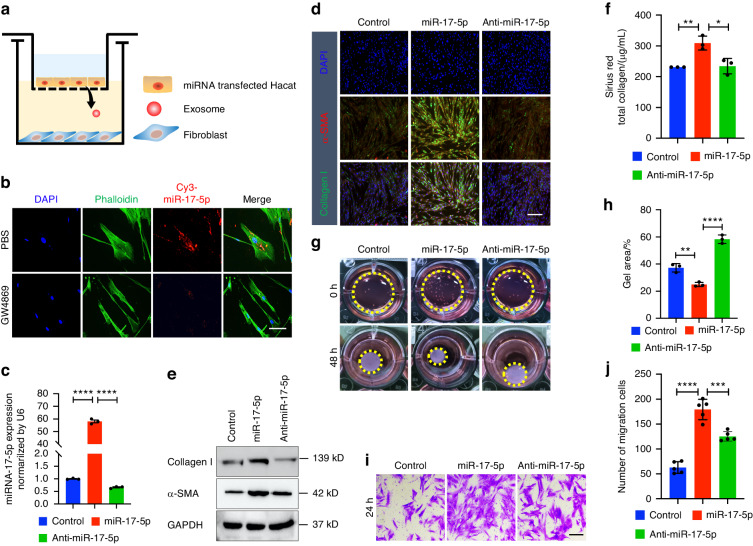
Exosomes derived from epithelial cells transfer miR-17-5p to fibroblast, which promotes myofibroblast differentiation. Control (epithelial cells transfected negative control), miR-17-5p (epithelial cells transfected mimic), Anti-miR-17-5p (epithelial cells transfected inhibitor), U6 or GAPDH was used as an internal control for qRT-PCR. **a** A transwell co-culture cell model with transfected epithelial cells (top well) and fibroblasts (bottom well). A 0.4-μm porous membrane is between the 2 wells, allowing the transmission of exosomes, but inhibiting direct contact between cells. **b** A co-culture assay to study the miRNA cargo from epithelial cells to fibroblasts. The epithelial cells were transfected with a Cy3-labeled miR-17-5p (red) and then treated with PBS or GW4869. Nuclei were counterstained with DAPI (blue). Scale bar: 50 μm. **c** The expression level of miR-17-5p in fibroblasts after 48 h co-culturing with epithelial cells transfected with miR-17-5p or anti-miR-17-5p. Immunostaining (**d**) and (**e**) Immunoblotting detection of collagen type I and α-SMA expression in fibroblast after 48 h co-culturing with epithelial cells. Scale bars, 100 μm. Sirius Red total collagen assay (**f**), collagen contraction assay (**g**, **h**) and transwell migration assay (**i**, **j**) were determined for fibroblast. *n* = 3–5 technical replicates, representative of two or three assays. Scale bars, 100 μm. Statistics: mean ± SEM, one-way ANOVA, *P < 0.05, **P < 0.01, ***P < 0.001, ****P < 0.000 1

To determine whether miR-17-5p is involved in the dysfunctional crosstalk between epithelial cells and fibroblasts, a coculture cell model was established. Briefly, we plated epithelial cells that were transfected with the Cy3-miR-17-5p mimic in the upper chamber, and we plated fibroblasts in the lower chamber (Fig. 3a). The coculture system was separated by a membrane with 0.4-μm pores, which allowed the transmission of microparticles, such as exosomes, but inhibited direct contact between the cells. After 36 h, we observed strong red fluorescence in fibroblasts, and this red fluorescence was lower when GW4869 was added to the coculture system (Fig. 3b). This phenomenon proved that miR-17-5p might be transferred from the epithelium to fibroblasts via exosomes. When epithelial cells were transfected with miR-17-5p (mimic) or anti-miR-17-5p (inhibitor) and cocultured with fibroblasts, the miR-17-5p levels were increased in the epithelium (Fig. S4a) and fibroblasts in the miR-17-5p group but decreased the anti-miR-17-5p group (Fig. 3c). Similarly, the levels of collagen type I and α-SMA were increased in fibroblasts that were cocultured with miR-17-5p-transfected epithelial cells but decreased in those cocultured with anti-miR-17-5p-transfected cells (Fig. S4b, c). Then, we investigated the role of epithelium-derived miR-17-5p in the differentiation phenotype of myofibroblasts. The levels of collagen type I and α-SMA were higher in fibroblasts in the miR-17-5p group (Fig. 3d, e). Moreover, collagen secretion (Fig. 3f), contraction (Fig. 3g, h) and migration (Figs. 3i, j) of fibroblasts were increased or decreased, respectively, when fibroblasts were cocultured with epithelial cells that were transfected with miR-17-5p or anti-miR-17-5p. This phenomenon proved that epithelial cell-derived exosomal miR-17-5p might participate in epithelium-fibroblast communication and induce the phenotypic transformation of fibroblasts.

### Exosomes-mediated transfer of the miR-17-5p target Smad7 directly promotes the myofibroblast differentiation phenotype

**Fig. 4:**
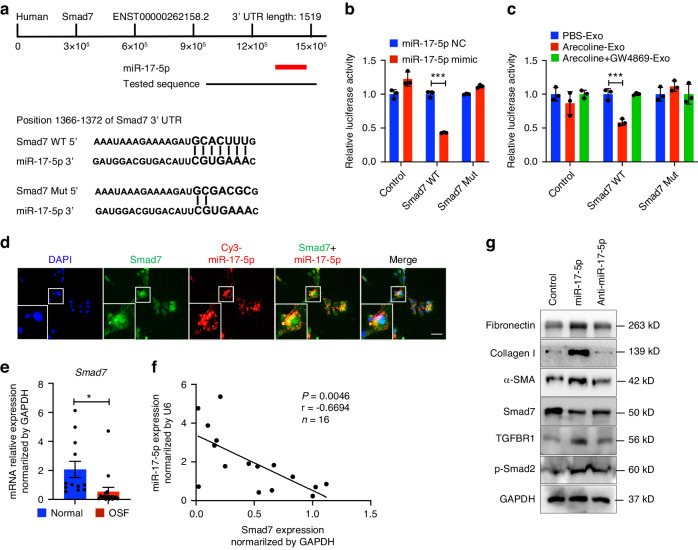
Exosomal miR-17-5p directly targets Smad7 in fibroblasts. **a** Schematic miR-17-5p predicted binding sites in the 3’ UTRs of Smad7 and the sequence of mutant UTRs. **b** Luciferase reporter assay was performed on HEK293T after co-transfected with the miR-NC or miR-17-5p mimic and the Smad7-wt plasmid or Smad7-mut plasmid. **c** Luciferase reporter was carried out in HEK293T transfected with Smad7-wt plasmid or Smad7-mut plasmid, then incubated with exosomes (50 μg/mL) derived from PBS-, Arecoline- and Arecoline+GW4869- treated epithelial cells for 48 h. **d** After transfection miR-17-5p in fibroblast, Immunostaining detection of miR-17-5p (red) and Smad7 (green) expression. Scale bars, 100 μm. **e** The expression level of Smad7 in normal (*n* = 12) and OSF (*n* = 17) tissues detected by qRT-PCR. **f** The relationship between relative Smad7 expression normalized to GAPDH and miR-17-5p expression normalized to U6. **g** Immunoblotting of Smad7, fibrotic markers and TGF-β path pathway. Statistics: mean ± SEM, unpaired, two-tailed Student’s *t* test, *P < 0.05, ***P < 0.001

To further explore the mechanism by which exosomes and miR-17-5p induce myofibroblast differentiation, we investigated the target genes that are involved in mediating the effect of miR-17-5p by using TargetScan (www.targetscan.org), MiRDB (www.mirdb.org) and MiRBase (www.mirbase.org). We found that the 3′ untranslated region (UTR) of Smad7 contained a binding site for miR-17-5p (Fig. 4a). Luciferase reporter assays were conducted using plasmids carrying Smad7 3’UTRs with wild-type or mutant miR-17-5p binding sites. HEK293T cells were cotransfected with a plasmid carrying the wild-type or mutant Smad7 3’UTR and the miR-17-5p mimic or control (NC). There was a significant reduction in luciferase activity when miR-17-5p and the wild-type Smad7 3’UTR were cotransfected into HEK293T cells. However, the luciferase activity was not affected when the binding site was mutated (Fig. 4b). Consistently, exosomes derived from Arecoline-treated epithelial cells also decreased the luciferase activity of the Smad7 3’UTR, but this effect was not observed in the PBS and exosomes inhibition groups (Fig. 4c). Then, we cocultured miR-17-5p-transfected epithelial cells with fibroblasts and found that miR-17-5p and its target Smad7 colocalized in fibroblasts (Fig. 4d). Notably, the expression of Smad7 was downregulated (Fig. S5a) and inversely correlated with the expression of miR-17-5p in tissues from clinical patients (Fig. 4e, f) and mice tissues (Fig. S5b, c). Furthermore, after fibroblasts were transfected with miR-17-5p for 48 h, Smad7 levels decreased, whereas inhibition of miR-17-5p reversed this effect and increases in TGFBR1 and p-Smad2 levels were evident (Fig. 4g). In addition, we observed that miR-17-5p increased the expression of Fibronectin, collagen type I and α-SMA in fibroblasts.

**Fig. 5:**
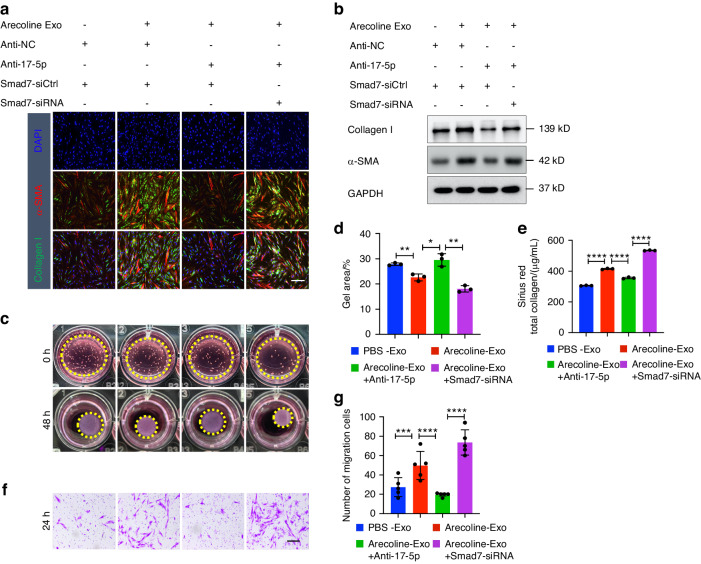
Exosomal miR-17-5p derived from Arecoline-treated epithelium confers fibroblast activation through Smad7. Fibroblasts were incubated with exosomes derived from PBS and Arecoline-treated epithelium, then transfection of Anti-15-5p or Smad7-siRNA for 48 h. Immunostaining (**a**) and Immunoblotting (**b**) detection of collagen type I and α-SMA expression in fibroblast. Scale bars, 100 μm. Collagen contraction assay (**c**, **d**), Sirius Red total collagen assay (**e**) and transwell migration assay (**f**, **g**) were determined for fibroblast. *n* = 3–5 technical replicates, representative of two or three assays. Statistics: mean ± SEM, one-way ANOVA, *P < 0.05, **P < 0.01, ***P < 0.001, ****P < 0.000 1

To determine whether Arecoline-treated epithelial cell-derived exosomal miR-17-5p promotes fibroblast differentiation by regulating Smad7, control, anti-miR-17-5p-transduced or Smad7 siRNA-transduced fibroblasts were exposed to exosomes derived from Arecoline-treated epithelium. The phenotype of the fibroblasts and the protein levels of collagen type I and α-SMA were determined. The results showed that downregulation of miR-17-5p blocked changes in fibrosis marker expression (Fig. 5a, b) and prevented the collagen contraction (Fig. 5c, d), secretion (Fig. 5e) and migration (Fig. 5f, g) of fibroblasts caused by exosomes derived from Arecoline-treated epithelial cells; however, silencing Smad7 attenuated the effect of the miR-17-5p inhibitor.

### miR-17-5p reverses the ubiquitination-mediated degradation of TGFBR1 mediated by the E3 ligase WWP1

Smad7 has been identified as a negative regulator that ameliorates TGF-β-mediated fibrogenesis. However, whether miR-17-5p interferes with Smad7-mediated TGF-β signaling is unclear. To this end, we transfected HEK293T cells with Smad7 and monitored the half-life of the TGFBR1 protein after treatment with 100 μg/mL cycloheximide (CHX, which is a protein synthesis inhibitor). Cell lysates were then collected within a specified period and analyzed by Immunoblotting. Compared to that in the control group, the protein level of TGFBR1 in the Smad7-transfected group decreased at each time point after CHX treatment (Fig. S6a, b). To further evaluate the effect of miR-17-5p on the expression of Smad7 and TGFBR1, we overexpressed miR-17-5p or Smad7 in HEK293T cells and fibroblasts and measured the expression of these proteins. Compared with those in the control group, miR-17-5p reduced the expression of Smad7 and increased the expression of TGFBR1, whereas overexpression of Smad7 had the opposite effects (Fig. S6c). Finally, we performed a Co-IP assay to further explore whether miR-17-5p acts as a scaffold to influence the binding of Smad7 to TGFBR1. We found that the association between Smad7 and TGFBR1 in HEK293T cells and fibroblasts was influenced by miR-17-5p overexpression or inhibition (Fig. S6d, e).

**Fig. 6:**
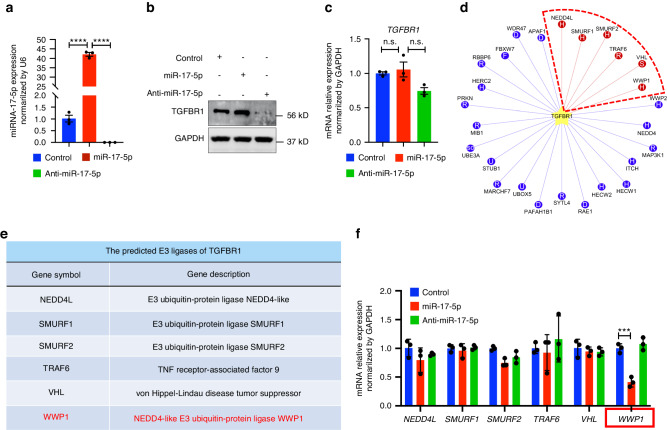
miR-17-5p inhibits the expression of E3 ubiquitin-protein ligase WWP1. **a** After transfected with miR-17-5p or anti-miR-17-5p for 48 h, the expression of miR-17-5p in fibroblasts was detected by qRT-PCR. Immunoblotting (**b**) and qRT-PCR (**c**) detection of TGFBR1 expression in fibroblasts transfected by miR-17-5p and anti-miR-17-5p. **d** UbiBrowser 2.0 online site predicts E3 ubiquitin ligase with TGFBR1 as substrate. **e** The annotation list for the predicted E3 ligases of TGFBR1. **f** qRT-PCR detection of E3 ubiquitin ligase (NEDD4L, SMURF1, SMURF2, TRAF6, VHL, WWP1) expression level in fibroblast transfected by miR-17-5p and anti-miR-17-5p. Statistics: mean ± SEM, one-way ANOVA, n.s.: no significance, ****P < 0.000 1

Numerous studies have confirmed that Smad7 inhibits TGF-β signaling by recruiting E3 ligases to TGFBR1, leading to the ubiquitination and degradation of the receptor.^29^ Therefore, we transfected miR-17-5p and anti-miR-17-5p into fibroblasts (Fig. 6a) and then analyzed the mRNA and protein expression of TGFBR1. Immunoblotting revealed that the overexpression of miR-17-5p significantly increased the expression of TGFBR1 in fibroblasts, while the inhibition of miR-17-5p had the opposite effect (Fig. 6b). However, qRT-PCR showed that the transcription of TGFBR1 was not affected by miR-17-5p (Fig. 6c). These results suggest that miR-17-5p may maintain the stability of TGFBR1 by inhibiting ubiquitination-dependent modifications and affecting the TGF-β pathway. To this end, we predicted TGFBR1-specific E3 ubiquitin ligases based on UbiBrowser 2.0 to determine the mechanism underlying miR-17-5p-mediated TGFBR1 ubiquitination (Fig. 6d).

**Fig. 7:**
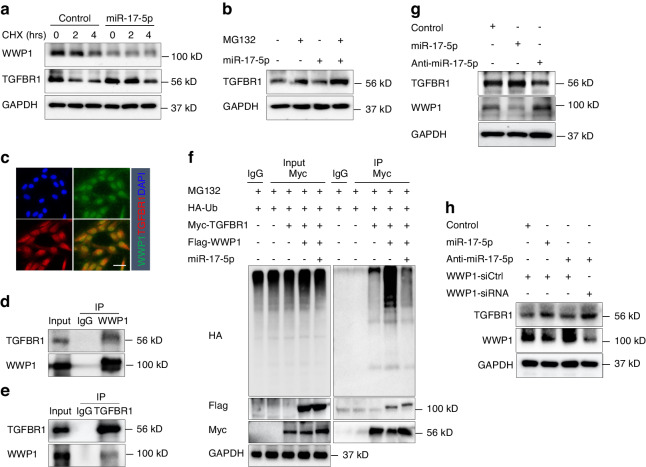
miR-17-5p inhibits WWP1-mediated degradation of TGFBR1 protein in fibroblasts. **a** Fibroblast transfected with miR-17-5p then incubated with cycloheximide (CHX, 100 μg/mL) for indicated time points. The protein levels of WWP1 and TGFBR1 were determined at indicated time points by Immunoblotting. **b** Fibroblasts transfected with miR-17-5p and anti-miR-17-5p then incubated with MG-132 (10 μM, 6 h). The protein levels of TGFBR1 were determined by Immunoblotting. **c** The expression localization of WWP1 (green) and TGFBR1 (red) in fibroblast was detected by Immunostaining. Scale bar: 50 mm. **d**, **e** Co-IP and Immunoblotting detected the interaction between WWP1 and TGFBR1 in fibroblast. **f** Co-IP and Immunoblotting detected the ubiquitination of TGFBR1 mediated by WWP1 and miR-17-5p overexpressed. **g**, **h** The expression of WWP1 and TGFBR1 protein levels in miR-17-5p overexpressed or inhibited in fibroblast was detected by Immunoblotting

We used qRT‒PCR to measure the expression levels of the top six E3 ubiquitination ligases (NEDD4L, SMURF1, SMURF2, TRAF6, VHL, and WWP1) in fibroblast after transfection with miR-17-5p (Fig. 6e). The results showed that only the WWP1 transcription levels were significantly inhibited (Fig. 6f). Next, we transfected miR-17-5p into fibroblasts for 48 h, after which the cells were treated with CHX. The cell lysates were then collected within a specified period and analyzed by Immunoblotting. Compared to the control group, miR-17-5p overexpression promoted WWP1 protein degradation but stabilized the protein expression of TGFBR1 (Fig. 7a). We also treated the cells with 10 μM MG132 (a specific inhibitor of a ubiquitin-binding protein) to explore the effect of miR-17-5p on TGFBR1 protein degradation. We observed increased TGFBR1 protein expression in cells that were treated with MG132 (Fig. 7b). Immunofluorescence revealed that WWP1 and TGFBR1 colocalized in the cytoplasm and nucleus (Fig. 7c). Exogenous and endogenous Co-IP experiments confirmed the interaction between WWP1 and TGFBR1 (Figs. 7d, e and S7a, b). Subsequently, HEK293T cells were cotransfected with Flag-WWP1 and HA-Ub plasmids, and TGFBR1 ubiquitination was detected by Immunoprecipitation and Immunoblotting. The results showed that WWP1 overexpression promoted TGFBR1 ubiquitination, while miR-17-5p reversed the WWP1-mediated ubiquitination and degradation of TGFBR1 (Fig. 7f). To further evaluate the effect of miR-17-5p on WWP1 expression, we overexpressed and inhibited miR-17-5p in fibroblasts and measured the protein expression levels of WWP1 and TGFBR1. The overexpression of miR-17-5p significantly decreased WWP1 protein expression and increased TGFBR1 protein expression compared with those in the control group, while the inhibition of miR-17-5p had the opposite effects (Fig. 7g). In addition, we confirmed that silencing WWP1 led to TGRBR1 protein accumulation in fibroblasts with inhibited miR-17-5p (Fig. 7h). These results suggest that the overexpression of miR-17-5p can inhibit the expression of the E3 ubiquitination ligase WWP1 and stabilize the expression of its substrate TGFBR1.

### WWP1 is involved in miR-17-5p-mediated myofibroblast differentiation

**Fig. 8:**
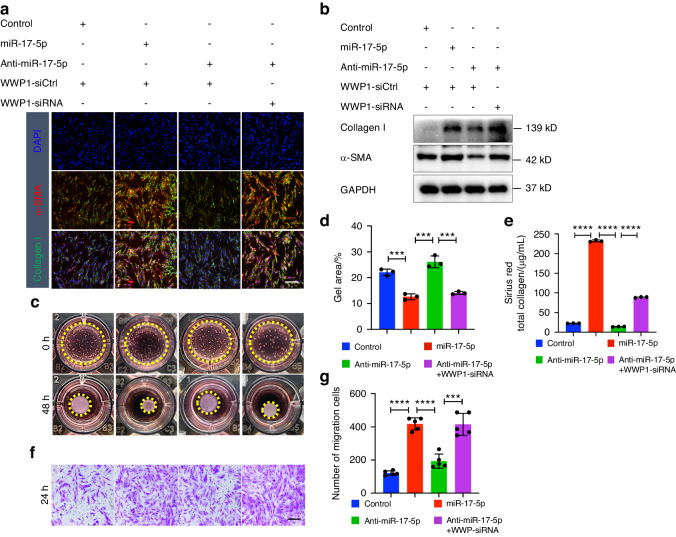
Down-regulated WWP1 expression can promote myofibroblast transformation. Fibroblasts were transfection of miR-17-5p, anti-15-5p or WWP1-siRNA for 48 h. Immunostaining (**a**) and Immunoblotting (**b**) detection of collagen type I and α-SMA expression in fibroblast. Scale bars, 100 μm. Collagen contraction assay (**c**, **d**), Sirius Red total collagen assay (**e**) and transwell migration assay (**f**, **g**) were determined for fibroblast. *n* = 3–5 technical replicates, representative of two or three assays. Statistics: mean ± SEM, one-way ANOVA, ***P < 0.001, ****P < 0.000 1

Additionally, we investigated whether WWP1 is involved in the phenotypic transformation of fibroblast. We transfected fibroblasts with miR-17-5p mimic or inhibitor and WWP1 siRNA, and then investigated the effects on the biological functions of the cells. The phenotype of fibroblast and protein levels of collagen type I and α-SMA were determined. Immunostaining and Immunoblotting showed that silencing WWP1 reversed the changes in fibrosis marker expression (Fig. 8a, b) and promoted the collagen contraction (Fig. 8c, d), secretion (Fig. 8e) and migration (Fig. 8f, g) of fibroblasts with inhibited miR-17-5p expression.

### Upregulation of miR-17-5p promotes OSF progression in mice

**Fig. 9:**
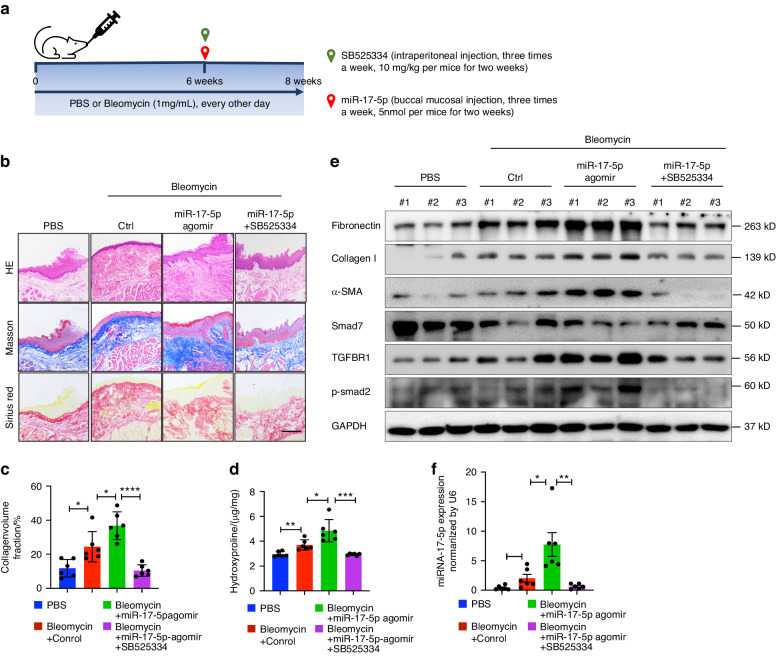
miR-17-5p induces collagen accumulation and promotes the progression of OSF by TGF-β in vivo. **a** Schematic overview of mouse experimental design. Mice were injected with 30 μL PBS or Bleomycin into the bilaterally buccal mucosa every other day for six weeks. Then, mice were simultaneously treated with miR-NC (Ctrl), miR-17-5p agomir and SB525334 for another two weeks. **b** HE, Masson and Sirius red staining of mice oral mucosa tissues. Scale bar, 200 μm. Quantification of Masson staining for collagen fraction. Scale bar, 200 μm. **d** Hydroxyproline content of mice oral mucosa tissues. **c** Quantification of Masson staining for skin tissues collagen fraction, *n* = 6 for each group. **e** Immunoblotting of fibrotic markers (Fibronectin, Collagen type I and α-SMA), Smad7, TGFBR1 and p-Smad2 pathway. **f** The levels of miR-17-5p of mice blood-derived exosomes. Statistics: mean ± SEM, *n* = 6 for each group, one-way ANOVA, *P < 0.05, **P < 0.01, ***P < 0.001, ****P < 0.000 1

To confirm the role of miR-17-5p in Bleomycin-induced OSF in mice, a miR-17-5p agomir was locally injected into the oral mucosa, and in another group, SB525334 (a TGF-β receptor inhibitor) was intraperitoneally injected simultaneously with Bleomycin (Fig. 9a). The results of histopathological section staining (Fig. 9b, c) and hydroxyproline (Fig. 9d) assays showed that mice that received the miR-17-5p agomir exhibited increased tissue collagen accumulation and volume. However, SB525334 effectively reversed the miR-17-5p-induced amplification of fibrosis. Immunoblotting revealed that in miR-17-5p agomir-treated mice, upregulated miR-17-5p induced the expression of fibronectin, collagen type I and α-SMA and activated the TGF-β pathway (Fig. 9e). Similarly, there higher expression of exosomal miR-17-5p was observed in the serum of the miR-17-5p agomir group than in that of the other groups (Fig. 9f). Moreover, the expression of Smad7 was decreased in mice that were treated with the miR-17-5p agomir, whereas SB525334 reversed this change in Smad7 expression. These experimental results were also validated in a mouse skin fibrosis model (Fig. S8). These data support our hypothesis that miR-17-5p can promote the development of fibrosis through the TGF-β pathway both in vitro and in vivo.

## Discussion

**Fig. 10:**
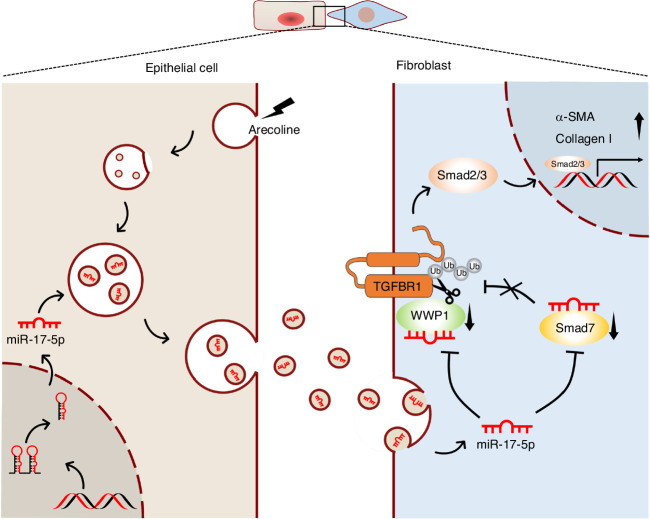
Working model showing that the Arecoline induces epithelial cells to release mir-17-5p enveloped exosomes, which could promote collagen contraction, secretion and migration after being absorbed by fibroblast. Mechanistically, it was demonstrated that miR-17-5p targets Smad7 and maintains TGFBR1 expression by inhibiting its E3 ubiquitination ligase WWP1, which promotes the expression of fibrosis hallmark genes

Our present study revealed a mechanism of exosome-mediated intercellular communication between epithelial cells and fibroblasts that promotes the development of OSF. Notably, we elucidated that this novel mechanism underlying myofibroblast differentiation is attributed to the regulation of TGF-β signaling by Arecoline-induced epithelial cells-derived exosomal miR-17-5p (Fig. 10).

OSF is mainly caused by chewing betel quid, and it is characterized by abnormal activation of epithelial cells that release saliva and various cytokines that promote immune cell activation, causing changes in the interaction between epithelial and mesenchymal cell types.^30^ Chewing betel quid results in the release of a variety of harmful toxic components and metabolic derivatives (Arecoline, Nitrosamine and reactive oxygen species, etc.), which drive the phenotypic alterations, including dysregulated expression of DNA, RNA and protein in epithelial cells, and may be involved in the regulation of fibrotic gene expression as a part of the pathogenesis of OSF.^8,31^ The differentiation of fibroblasts into myofibroblasts and the accumulation of extracellular matrix are defining features of tissue remodeling during OSF.^32^ Recent studies have shown that DNA methyltransferase 3 A (DNMT3A) can increase the viability, invasion and migration of oral fibroblasts by promoting methylation of the VHL promoter.^33^ Arecoline induces persistent activation of fibroblast by promoting the expression of the transcription factor Snail. Moreover, Snail forms a positive feedback loop with downstream IL-6 to regulate the areca nut-associated myofibroblast differentiation.^34^ On the other hand, arecoline can promote the IGF-1R-dependent binding of ZEB1 and Slug to the E-box region of α-SMA and collagen type I, respectively, which promote the differentiation of myofibroblasts.^35^ Our previous study revealed that Arecoline can activate the BRAF/MEK1 signaling pathway in epithelial cells, which ultimately promotes epithelial-mesenchymal transition.^36^ However, how epithelial cells drive the differentiation of fibroblasts to participate in the development of fibrosis is unclear.

In recent years, exosomes have been used as potential natural carriers to transport active molecules to recipient cells and play a biological role in fibrosis.^37^ For example, cigarette smoke triggers the modification of exosomes components in bronchial epithelial cells and mediates myofibroblast differentiation via pVHL/HIF-1α signaling.^38^ Moreover, ischemia perfusion injury can induce the release of exosomes from tubular epithelium cells that negatively regulate the expression of suppressor of cytokine signaling 1 to promote renal fibrosis.^39^ Here, we found that Arecoline induces the release of exosomes from epithelial cells and that these exosomes promote myofibroblast differentiation after they are taken up by fibroblasts. These observations suggest that stimulation during areca chewing disrupts the epithelial barrier and alters the composition of exosomes, which in turn disrupts the balance between epithelial cells and fibroblasts, thereby inducing tissue remodeling.

Among many bioactive molecules, miRNAs are of tremendous interest in the field of exosome research because of their powerful ability to regulate gene expression.^40^ MiRNAs can be encapsulated in these phospholipid bilayer vesicles to prevent their degradation, then modify the expression of target genes and regulate signal transduction and biological processes.^41^ Indeed, there are some reports that miRNAs are involved in the development of OSF. For example, miR-424 is aberrantly overexpressed in OSF tissues, and the suppression of miR-424 markedly reduces collagen contractility, migration ability and downregulates the expression of fibrosis markers via directly binding to TGIF2.^42^ The profibrotic molecule miR-155 is involved in the pathogenesis of oxidative stress-associated OSF through the regulation of RPTOR. In addition, some miRNAs possess the function of inhibiting fibrogenesis.^43^ The Arecoline-induced myofibroblast activities are abolished by the overexpression of miR-200b/c in fibroblasts, which demonstrates that the miR-200b-mediated decrease in ZEB1/2 leads to the downregulation of α-SMA and vimentin.^44^ Finally, overexpressed miR-29b binds directly to the 3’UTR of collagen type I and subsequently ameliorates myofibroblast phenotypes. However, these studies are not sufficient to explain the source of these fibrosis-related miRNAs and the specific mechanisms by which they perform their functions.

In this study, miRNA sequencing revealed that miR-17-5p was more highly expressed in arecoline-induced epithelial cell-derived exosomes than in PBS-induced epithelial cell-derived exosomes. The miR-17-5p was shown to be a “long-lived miR” in an animal model, which extended the lifespan of transgenic miR-17-5p overexpressing mice.^45^ In addition, the overexpression of miR-17-5p in mouse cardiac fibroblasts enhances cell survival under oxidative stress conditions and increases proliferation by targeting tissue inhibitors of metalloproteinases, leading to matrix remodeling after infarction.^46^ Our results showed that arecoline-induced epithelial cell-derived exosomal miR-17-5p facilitated myofibroblast differentiation and upregulated the expression of hallmarks of fibrosis. However, transfection of anti-miR-17-5p into epithelial cells partially blocked the profibrotic effect of exosomes. These results indicated that the delivery of miR-17-5p was depended on exosomes. Mechanistically, exosomal miR-17-5p drive myofibroblast differentiation by targeting the downstream TGF-β pathway of Smad7 inactivation and enhancing the expression of fibrosis hallmarks.

TGF-β1 is considered the main pathogenic factor or “master regulator” that drives organ fibrosis.^47^ TGF-β1 acts as a typical signaling pathway involving the phosphorylation and activation of Smad2/3 by the TGF-β receptor, which is involved in physiological processes such as cell differentiation and the immune response.^48^ Smad7 is a negative feedback inhibitor of canonical TGF-β/Smad signaling. Previous studies have shown that Smad7 knockout mice exhibit increased susceptibility to renal fibrosis, whereas overexpression of Smad7 through gene transfer ameliorates renal fibrosis.^49^ In the present study, we demonstrated that exosomal miR-17-5p promotes the expression of genes downstream of TGF-β by targeting the 3′UTR of Smad7. Moreover, we verified that Smad7 is expressed at low levels in tissues from OSF patients and mouse models. Mechanistically, Smad7 serves as a scaffold to recruit the E3 ubiquitin ligase to the TGF-β receptor or Smad complex to facilitate receptor polyubiquitination and complex degradation.^50,51^ Furthermore, Smad7 itself can also be degraded through the ubiquitination pathway or regulated by deubiquitinating enzymes to maintain protein stability.^52^ However, the role of miR-17-5p in the regulation of Smad7-mediated TGFBR1 ubiquitination remains unclear. Previous studies reported that miR-769-5p facilitates cisplatin resistance and progression in cancer cells by targeting CASP9 and promoting the ubiquitination-mediated degradation of p53.^53^ Our results proved that miR-17-5p exerts a regulatory effect that results in an imbalance between the downregulation of Smad7 and the activation of TGFBR1. In addition, we also proved that miR-17-5p plays an important role in TGFBR1 protein ubiquitination by degrading the E3 ubiquitin ligase WWP1, thereby revealing a novel miRNA- mechanism underlying the ubiquitination of the TGRBR1 protein.

Under normal cellular physiological conditions, exosomes secretion can be understood as a process by which cells maintain homeostasis, as exosome secretion can remove harmful components in response to stimuli from the external environment.^54^ During the betel quid chewing, we hypothesize that epithelial cell exposure to stressors can modify the composition of exosomes to remodel the microenvironment to promote self-protection mechanisms and to target the differentiation of fibroblasts through cell-to-cell communication. On the other hand, circulating exosomes are potentially useful as disease biomarkers for they carry specific proteins and nucleic acids.^55^ Indeed, here we determined the role of miR-17-5p in a Bleomycin-induced mouse model of OSF. Consistent with the cell culture results, miR-17-5p expression was upregulated in oral mucosal tissues and serum exosomes of Bleomycin-treated mice, and in these mice, Smad7 was significantly downregulated and TGF-β signaling was significantly activated. In mice, inhibition of the TGF-β receptor attenuated miR-17-5p induced collagen accumulation and tissue remodeling. Although we focused primarily on the composition of miRNAs in exosomes, this approach is equally important for analyzing the details of other exosomes components, such as fibrosis-related proteins, and long or circular noncoding RNAs, that may influence the cellular phenotype of and epigenetic changes in fibroblasts. In any event, our present study showed that miRNAs or exosomal miRNAs have the potential to be biomarkers of OSF, although technical and scientific problems must be overcome before their clinical application.

In summary, our data reveal a novel mechanism by which tissue remodeling is regulated by epithelium-derived exosomal miR-17-5p in response to Arecoline exposure. After the uptake of exosomes by fibroblasts, miR-17-5p can target Smad7 and activate the TGF-β pathway as well as inhibit the expression of the TGFBR1-specific E3 ubiquitination ligase WWP1, which promotes the expression of hallmarks of fibrosis, inducing the myofibroblast differentiation phenotype. These findings suggest that chewing betel quid alters exosomal compositions and identify epithelial cell-derived miR-17-5p as a paracrine mediator of myofibroblast differentiation that has potential to be a target for treating OSF.

## Materials and methods

### Tissue samples and clinical specimens

This study collected oral mucosa tissues from 15 healthy individuals and 17 OSF patients who underwent oral outpatient surgery at Xiangya Stomatological Hospital, Central South University, Hunan, China. OSF tissue samples were derived from the patient’s first visit with no history of treatment and had definitely pathological diagnosis by two experienced pathologists. Written informed consent was obtained from all patients and the study was approved by the Protection of Human Subjects Committee of Xiangya Stomatological Hospital, Central South University (No. 20220102).

### Cell culture and treatment

Primary buccal oral mucosa fibroblast was obtained from healthy individuals who did not have areca quid chewing habits, which were isolated and cultivated by tissue adherence method. The primary fibroblast, HaCat cell lines and HEK293T cells were cultured in Dulbecco’s Modified Eagle Medium (DMEM) (Gibco, Grand Island, NY, USA) containing 10% bovine calf serum (Sigma-Aldrich, MO, USA) under at 37 °C with 5% CO_2_. Plasmid transfection using Lipo8000™ transfection reagent was carried out following the manufacturer’s instructions (Beyotime, Shanghai, China). After reaching 70%–80% confluence, HaCat cells were washed with PBS and then grown in DMEM without FBS and exposed to Arecoline (Sigma-Aldrich, MO, USA) for 24 h. In a co-culture model, PBS or Arecoline-treated HaCat cells were plated on 0.4-μm pore, hanging cell culture chambers (Millipore, MA, USA), and fibroblast cells were seeded on 12- or 6-well plates.

### Exosome isolation and characterization

Exosomes were isolated from the exosomes-free culture medium of PBS or Arecoline-treated HaCat cells by differential centrifugation. The culture medium was first centrifuged at 300 × *g* for 100 min and then at 2 000 × *g* for 20 min at 4 °C. The supernatant was then through a 0.22-μm filter (Millipore, MA, USA) to remove cell debris and other larger vesicles. Finally, the supernatant was centrifuged at 100 000 × *g* for 90 min. The collected exosomes were resuspended in PBS and subjected to the next experiments. Meanwhile, the isolated exosomes from the culture medium and blood serum were collected by exosome isolation reagent (Ecotop Scientific, Guangzhou, China). The size distribution and concentration of exosomes were analyzed by nanoparticle tracking analysis (NTA) (ZetaView, Particle Metrix, Germany). The expression of the exosomal markers CD9, CD63 and CD81 (exosome panel, ab275018, Abcam) was assessed by Immunoblotting analysis.

### DiI staining for exosomes

Exosomes derived from epithelial cells were labeled with DiI cell membrane red fluorescent probe (Beyotime, Shanghai, China) according to the manufacturer’s protocol with minor modifications. Briefly, exosomes were suspended in PBS and mixed with diluted DiI (10 μmol/L, for labeling of cell membranes) and incubated at room temperature. After 30 min, the labeled exosomes were finally washed with PBS, and they were resuspended for uptake experiments.

### RNA extraction and real-time PCR

Total RNA from cultured cells and tissues of mice was isolated by use of Trizol reagent (Invitrogen, CA, USA) according to the manufacturer’s recommendations. Extraction of miRNA from exosomes was accomplished using a commercial RNAeasy small RNA isolation Kit (Beyotime, Shanghai, China). The purified RNA was eluted with RNase-free water and stored at –80 °C until analysis. MiRNA qRT-PCR synthesis and primer sets kits (Sangon Biotech, Shanghai, China) specific for miR-17-5p and U6 snRNA were used to measure the levels of miRNAs. The U6 snRNA and GAPDH were used as endogenous controls. Real-time PCR was performed by use of SYBR Green (Vazyme, Jiangsu, China) with the Applied Biosystems QuantStudio 5 (Thermo Fisher, MA, USA). To analyze the qRT-PCR results for experiments, the 2^−ΔΔCt^ method was used. The PCR primers are shown in Supplementary Table 1.

### Immunoprecipitation, Immunoblotting and Immunostaining

Cell or tissue samples were lysed on ice by RIPA buffer mixed with protease and phosphatase inhibitor cocktails (Beyotime, Shanghai, China). The proteins were then quantitatively analyzed by a BCA Protein Assay Kit (23225, Thermo Fisher, MA, USA). The proteins were then separated by 8%–10% SDS-PAGE gel and transferred to PVDF membranes. Membranes were then incubated overnight at 4 °C with a primary antibody for COL1A1 (1:2 000, 67288, Proteintech), α-SMA (1:2 000, 14395, Proteintech), Smad7 (1:2 000, 25840, Proteintech), TGFBR1 (1:2 000, A0708, ABclonal), WWP1 (1:1 000, sc-390897, Santa Cruz Biotechnology), Fibronectin (1:2 000, 66042, Proteintech), Phospho-Smad2 (Ser465/Ser467) (1:2 000, #18338, Cell Signaling Technology), GAPDH (1:2 000, 5174 S, Cell Signaling Technology). The membranes were then incubated with a 1:5 000 dilution of horseradish peroxidase-conjugated goat anti-rabbit and anti-mouse antibodies (Cell Signaling Technology) for 1 h at room temperature and images were visualized using a chemiluminescent imaging system (BIO-RAD, USA).

The interaction between proteins was verified by immunoprecipitation. In brief, cell lysates were incubated with indicated antibodies and Protein A/G Agarose (Beyotime, Shanghai, China) at 4 °C overnight. The immunocomplex was washed 5 times, and boiled in 2× SDS loading buffer for 10 min. The coprecipitation was performed on SDS-PAGE gel and blotted with the specific antibody.

For immunofluorescence staining, cells were cultured on coverslips washed 3 times with PBS, and fixed with 4% paraformaldehyde for 30 min. The glass coverslips permeabilized with 0.5% Triton X-100 at room temperature for 20 min, then blocked with 3% BSA in PBS for 1 h at room temperature and incubated with primary antibody at 4 °C overnight. The coverslips were washed 3 times with PBS and incubated with the corresponding fluorescent secondary antibody conjugated with fluorochrome for 1 h at 37 °C. The cell nuclei were stained with DAPI. Finally, cell images were observed and captured with fluorescence microscopy (Leica, Wetzlar, Germany).

### Sirius red collagen detection

Collagen content in cell supernatant was detected using the Sirius Red Total Collagen Detection Kit (#9062, Chondrex, WA, USA) according to the manufacturer’s instructions. Briefly, the fibroblast culture medium was incubated with concentrating solution (#90626, Chondrex, WA, USA) at 4 °C overnight. After centrifugation at 10 000 r/min for 3 min, the precipitate was collected and dissolved in 0.05 mol/L acetic acid. Then the blank, standard and sample solutions were incubated with Sirius Red Solution. Finally, removed the supernatant and dissolved in the extraction buffer. Read the optical density values at 510–550 nm and the collagen concentration (μg/mL) in test samples was calculated using regression analysis.

### Three-dimensional collagen gels

The treated fibroblast resuspended in 5× DMEM were mixed with 3 mg/mL rat tail type I collagen (Cellmatrix, Nitta Gelatin, Japan) at a ratio of 2:1. The mixture was seeded in 24-well plates at a cell density of 5 × 10^4^per well. After collagen coagulation for 30 min at 37 °C, the edges of the gel separated from the wall of the well, and 0.5 mL culture medium was added. The gels were photographed by 48 h and the gel area was measured using ImageJ software (NIH, Bethesda, MD, USA).

### Animal studies

Male BALB/c mice at 4 weeks of age were purchased from Hunan SJA Laboratory Animal Co. Ltd. (China) and housed in animal facilities at the Department of Laboratory Animals of Central South University. Animals were treated humanely and with regard for alleviation of suffering according to a protocol approved by the Institutional Animal Care and Use Committee of the School of Basic Medicine Science, Central South University (2022-KT66). To verify the expression of miR-17-5p in tissues, mice were exposed to PBS or Bleomycin 1 mg/mL (Yeasen, Shanghai, China) as described previously. Briefly, twelve mice were randomly divided into two groups. Every other day, we used a 29-gauge insulin syringe (Ultra-Fine, BD) to inject drug treatment into the bilateral buccal mucosa (30 μL) and back skin (50 μL) of the mouse for 6 weeks. For inhibition of miR-17-5p profibrotic effects, twenty-four mice were divided into four groups (PBS, Bleomycin + control, Bleomycin + miR-17-5p agomir, Bleomycin + miR-17-5p agomir + SB525334). After 6 weeks of Bleomycin treatment, miR-17-5p and TGF-β inhibitor SB525334 were administered concurrently for 2 weeks, respectively. Mice in the miR-17-5p agomir and control groups (5nmol, RiBoBio, China) were dosed with buccal mucosa injection 3 times a week for 2 weeks. SB525334 compound was dosed with intraperitoneal injection (10 mg/kg, SB525334, Selleck) daily for 2 weeks, After the mice were sacrificed, the buccal mucosa, back skin and blood sample obtained for the subsequent experiments.

### Dual-luciferase reporter assay

TargetScan was used to predict the binding sites for miR-17-5p in Smad7. The 3’UTR sequence of Smad7, which was predicted to harbor the miR-17-5p seed region, or a mutant sequence, was inserted into the SacI and XbaI sites of the pmirGLO luciferase vector (Sangon Biotech, Shanghai, China). HEK293T cells were seeded in 24-well plates and co-transfected by DNA transfection reagent (Neofect biotech, Beijing, China) with pmirGLO vectors containing wild-type or mutant 3’-UTR of Smad7 and miR-17-5p mimics or miR-17-5p mimic-NC to examine the miRNA binding ability. About 48 h later, the cells were washed and lysed with the lysis buffer from the Dual-Luciferase Reporter Gene Assay Kit (Sangon Biotech, Shanghai, China) according to the manufacturer’s instructions. Finally, the luciferase and renilla activity was detected by a chemiluminescence instrument (Promega, USA). Relative luciferase activity was normalized with renilla luciferase activity and then compared with those of the respective control.

### Pathological tissue staining

The obtained buccal mucosa and skin tissue of the mouse was fixed (4% paraformaldehyde), dehydrated, and embedded in paraffin and sectioned for staining with HE, Masson’s trichrome (NJJCBIO, Nanjing, China) and Sirius red (Solarbio, Beijing, China) to assess the degree of fibrosis. The experimental procedures of tissue section staining were according to the reagent instructions. The collagen volume fraction area was read by ImageJ software.

## Statistical analysis

Data are expressed as the mean ± standard error of the mean (SEM). All of the statistical analyses were assessed by GraphPad Prism software. Student’s t test was used to determine the significance of the difference between the two groups, and the ANOVA was performed to evaluate the statistical differences among groups. Pearson’s correlation coefficient was used for correlation analysis. For all tests, a p value less than 0.05 was considered significantly different (*P < 0.05, **P < 0.01, ***P < 0.001, ****P < 0.000 1).

### Supplementary information

Revision Supplementary Information

Supplementary Figure 1

Supplementary Figure 2

Supplementary Figure 3

Supplementary Figure 4

Supplementary Figure 5

Supplementary Figure 6

Supplementary Figure 7

Supplementary Figure 8

## Data Availability

The data presented in this study are available upon request from the corresponding author.

## References

[1] Wynn TA, Ramalingam TR (2012). Mechanisms of fibrosis: Therapeutic translation for fibrotic disease. Nat. Med..

[2] Zhao M (2022). Targeting fibrosis, mechanisms and cilinical trials. Signal Transduct Target Ther..

[3] Henderson NC, Rieder F, Wynn TA (2020). Fibrosis: From mechanisms to medicines. Nature.

[4] Yuwanati M (2023). Prevalence of oral submucous fibrosis among areca nut chewers: A systematic review and meta-analysis. Oral Dis..

[5] Qin X (2023). Oral submucous fibrosis: Etiological mechanism, malignant transformation, therapeutic approaches and targets. Int. J. Mol. Sci..

[6] Ray JG, Chatterjee R, Chaudhuri K (2019). Oral submucous fibrosis: A global challenge. Rising incidence, risk factors, management, and research priorities. Periodontol 2000.

[7] Sharma M (2020). Loss of oral mucosal stem cell markers in oral submucous fibrosis and their reactivation in malignant transformation. Int. J. Oral Sci..

[8] Ko AMS, Tu HP, Ko YC (2023). Systematic review of roles of arecoline and arecoline N-oxide in oral cancer and strategies to block carcinogenesis. Cells.

[9] Plikus MV (2021). Fibroblasts: Origins, definitions, and functions in health and disease. Cell.

[10] Lynch MD, Watt FM (2018). Fibroblast heterogeneity: Implications for human disease. J. Clin. Invest..

[11] Wei K, Nguyen HN, Brenner MB (2021). Fibroblast pathology in inflammatory diseases. J. Clin. Invest.

[12] Buechler MB, Fu W, Turley SJ (2021). Fibroblast-macrophage reciprocal interactions in health, fibrosis, and cancer. Immunity.

[13] Lendahl U, Muhl L, Betsholtz C (2022). Identification, discrimination and heterogeneity of fibroblasts. Nat. Commun.

[14] Pakshir P (2020). The myofibroblast at a glance. J. Cell Sci..

[15] Ogawa M, LaRue AC, Drake CJ (2006). Hematopoietic origin of fibroblasts/myofibroblasts: Its pathophysiologic implications. Blood.

[16] Schuster R (2023). The role of myofibroblasts in physiological and pathological tissue repair. Cold. Spring Harb. Perspect. Biol..

[17] Gibb AA, Lazaropoulos MP, Elrod JW (2020). Myofibroblasts and fibrosis: Mitochondrial and metabolic control of cellular differentiation. Circ. Res.

[18] Hinz B, Lagares D (2020). Evasion of apoptosis by myofibroblasts: a hallmark of fibrotic diseases. Nat. Rev. Rheumatol.

[19] Waasdorp M (2021). The bigger picture: Why oral mucosa heals better than skin. Biomolecules.

[20] Farid H (2022). Oral manifestations of Covid-19-A literature review. Rev. Med. Virol.

[21] Nikoloudaki G, Creber K, Hamilton DW (2020). Wound healing and fibrosis: A contrasting role for periostin in skin and the oral mucosa. Am. J. Physiol. Cell Physiol..

[22] Katsuno Y, Derynck R (2021). Epithelial plasticity, epithelial-mesenchymal transition, and the TGF-beta family. Dev Cell.

[23] Théry C, Zitvogel L, Amigorena S (2002). Exosomes: Composition, biogenesis and function. Nat. Rev. Immunol.

[24] Isaac R (2021). Exosomes as mediators of intercellular crosstalk in metabolism. Cell Metab..

[25] Li SR (2021). Tissue-derived extracellular vesicles in cancers and non-cancer diseases: Present and future. J. Extracell Vesicles.

[26] Xie C (2019). The role of extracellular vesicles from different origin in the microenvironment of head and neck cancers. Mol. Cancer.

[27] Mathieu M (2019). Specificities of secretion and uptake of exosomes and other extracellular vesicles for cell-to-cell communication. Nat. Cell Biol..

[28] Kalluri R, LeBleu VS (2020). The biology, function, and biomedical applications of exosomes. Science.

[29] Gao S (2022). PKM2 promotes pulmonary fibrosis by stabilizing TGF-beta1 receptor I and enhancing TGF-beta1 signaling. Sci. Adv..

[30] Xu HQ (2023). Fibrotic matrix induces mesenchymal transformation of epithelial cells in oral submucous fibrosis. Am. J. Pathol.

[31] Hu X (2022). Overexpression of DEC1 in the epithelium of OSF promotes mesenchymal transition via activating FAK/Akt signal axis. J. Oral Pathol Med.

[32] Li, M. et al. Fibroblast activating protein promotes the proliferation, migration, and activation of fibroblasts in oral submucous fibrosis. *Oral Dis*. Epub ahead of print. (2023). 10.1111/odi.14602 37357365

[33] Kuang, H. et al. DNA methyltransferase 3A induces the occurrence of oral submucous fibrosis by promoting the methylation of the von Hippel-Lindau. *Oral Dis*. Epub ahead of print. (2023). 10.1111/odi.14725 37743610

[34] Peng CY (2020). Positive feedback loop of SNAIL-IL-6 mediates myofibroblastic differentiation activity in precancerous oral submucous fibrosis. Cancers (Basel).

[35] Fang CY (2019). Slug mediates myofibroblastic differentiation to promote fibrogenesis in buccal mucosa. J. Cell Physiol.

[36] Xie C (2022). Identification of a BRAF/PA28gamma/MEK1 signaling axis and its role in epithelial-mesenchymal transition in oral submucous fibrosis. Cell Death Dis..

[37] Dinh PUC (2020). Inhalation of lung spheroid cell secretome and exosomes promotes lung repair in pulmonary fibrosis. Nat. Commun.

[38] Xu H (2018). Exosomal microRNA-21 derived from bronchial epithelial cells is involved in aberrant epithelium-fibroblast cross-talk in COPD induced by cigarette smoking. Theranostics.

[39] Zhou X (2021). Tubular cell-derived exosomal miR-150-5p contributes to renal fibrosis following unilateral ischemia-reperfusion injury by activating fibroblast in vitro and in vivo. Int. J. Biol. Sci..

[40] Garcia-Martin R (2022). MicroRNA sequence codes for small extracellular vesicle release and cellular retention. Nature.

[41] Zhao S (2020). Tumor-derived exosomal miR-934 induces macrophage M2 polarization to promote liver metastasis of colorectal cancer. J. Hematol. Oncol.

[42] Chou MY (2023). MiR-424/TGIF2-mediated pro-fibrogenic responses in oral submucous fibrosis. Int. J. Mol. Sci..

[43] Chou MY (2022). Depletion of miR-155 hinders the myofibroblast activities and reactive oxygen species generation in oral submucous fibrosis. J. Formos Med. Assoc..

[44] Liao YW (2018). miR-200b ameliorates myofibroblast transdifferentiation in precancerous oral submucous fibrosis through targeting ZEB2. J. Cell Mol. Med.

[45] Du WW (2014). miR-17 extends mouse lifespan by inhibiting senescence signaling mediated by MKP7. Cell Death Dis..

[46] Du WW (2015). The microRNA miR-17-3p inhibits mouse cardiac fibroblast senescence by targeting Par4. J. Cell Sci..

[47] Peng D (2022). Targeting TGF-beta signal transduction for fibrosis and cancer therapy. Mol. Cancer.

[48] Meng X, Nikolic-Paterson DJ, Lan HY (2016). TGF-beta: The master regulator of fibrosis. Nat. Rev. Nephrol.

[49] Chung ACK (2009). Disruption of the Smad7 gene promotes renal fibrosis and inflammation in unilateral ureteral obstruction (UUO) in mice. Nephrol Dial Transpl..

[50] Aragón E (2012). Structural basis for the versatile interactions of Smad7 with regulator WW domains in TGF-beta Pathways. Structure.

[51] Fukasawa H (2004). Down-regulation of Smad7 expression by ubiquitin-dependent degradation contributes to renal fibrosis in obstructive nephropathy in mice. Proc. Natl. Acad. Sci. USA.

[52] Kit Leng Lui S (2017). USP26 regulates TGF-beta signaling by deubiquitinating and stabilizing SMAD7. EMBO Rep..

[53] Jing X (2022). Exosome-transmitted miR-769-5p confers cisplatin resistance and progression in gastric cancer by targeting CASP9 and promoting the ubiquitination degradation of p53. Clin. Transl. Med.

[54] Takahashi A (2017). Exosomes maintain cellular homeostasis by excreting harmful DNA from cells. Nat. Commun.

[55] Chen G (2018). Exosomal PD-L1 contributes to immunosuppression and is associated with anti-PD-1 response. Nature.

