# Supplementary material for: Exosomal miR-17-5p derived from epithelial cells is involved in aberrant epithelium-fibroblast crosstalk and induces the development of oral submucosal fibrosis
Source: Int J Oral Sci. 2024 Jun 20;16:48. doi: 10.1038/s41368-024-00302-2 (PMC11187069; doi:10.1038/s41368-024-00302-2)
Supplement: Supplementary file 1 — Revision Supplementary Information [file 41368_2024_302_MOESM1_ESM.docx]

**Supplementary Information**

**Supplementary Figure 1** Isolation and identification of exosomes derived from epithelial cells. PBS-Exo (exosomes derived from epithelial cells treated with PBS), Arecoline-Exo (exosomes derived from epithelial cells treated with Arecoline), Arecoline+GW4869-Exo (exosomes derived from epithelial cells treated with Arecoline and GW4869). **a** Exosomes in the supernatants of epithelial cells treated with PBS or Arecoline (20 μg/mL, 48 h) were collected by ultracentrifugation. **b** Transmission electron microscopy (TEM, top panel) images of double-membrane exosomes purified from PBS and Arecoline groups supernatants. Scale bars, 100 nm. Exosomes detection by nanoparticle-tracking analysis (NTA, bottom panel). The most common particle diameter is on average 139 nm (PBS-Exo) and 140 nm (Arecoline-Exo). **c** The hallmarks of exosomes (TSG101, CD9 and CD81) and cellular cytoplasmic proteins (Calnexin and GAPDH) were detected by Immunoblotting. **d** Expression levels of exosomes hallmarks in PBS-, Arecoline-, and GW4869 treated groups by Immunoblotting.

**Supplementary Figure 2** Epithelial-derived exosomes promotes the expression of collagen type I and α-SMA in fibroblasts. PBS-Exo (exosomes derived from epithelial cells treated with PBS), Arecoline-Exo (exosomes derived from epithelial cells treated with Arecoline), Arecoline+GW4869-Exo (exosomes derived from epithelial cells treated with Arecoline and then treated with GW4869). **a-c** Immunoblotting (a) was performed and relative protein levels (b, c) of collagen type I and α-SMA were determined for fibroblasts. **d** Immunoblotting of collagen type I and α-SMA in PBS-, Arecoline- and Arecoline+GW4869-Exo. Statistics: mean ± SEM, unpaired, two-tailed Student’s t-test (b, c), n.s.: no significance, **p < 0.01, ****p < 0.0001.

**Supplementary Figure 3** The expression of miR-17-5p was up-regulated in the OSF mice model. **a, f** Schematic overview of mouse OSF (a) and skin fibrosis (f) experimental design. Mice were injected with 30 μL drug into the bilaterally buccal mucosa or 50 μL drug into the back skin every other day, for six weeks. **b, g** Scanning electron microscope image of the mice oral mucosa (b) and skin (g) lamina propria. Scale bars, 10 μm (left panel), 5 μm (right panel). **c, h** HE, Masson and Sirius red staining of oral (c) and skin tissues (h) after PBS or Bleomycin exposure. Scale bar, 200 μm. **d, i** Quantification of Masson staining for oral (d) and skin (i) tissues collagen fraction, n=6 for each group. **e, j** Hydroxyproline content of mice oral mucosa (e) and skin tissues (j) treated after PBS or Bleomycin exposure, n=6 for each group. **k** Immunoblotting of Fibronectin, Collagen type I and α-SMA in mice oral mucosa (left) and skin tissues (right). **l-o** The levels of miR-17-5p of mice blood-derived exosomes (l), oral mucosal tissues (m) and skin tissues (o) after PBS or Bleomycin exposure, n=6 for each group. Statistics: mean ± SEM, unpaired, two-tailed Student’s t-test, *p < 0.05, **p < 0.01, ***p < 0.001.

**Supplementary Figure 4** The expression level of miR-17-5p was detected by qRT-PCR. **a** The expression levels of miR-17-5p in epithelial cells transfected with miR-17-5p or anti-miR-17-5p. **b, c** The expression level of miR-17-5p in fibroblasts after 48 h co-culturing with epithelial cells transfected with miR-17-5p or anti-miR-17-5p. Statistics: mean ± SEM, one-way ANOVA, *p < 0.05, **p < 0.01, ****p < 0.0001.

**Supplementary Figure 5** The expression relationship between Smad7 and miR-17-5p. **a** The expression level of Smad7 in fibroblast transfected with miR-17-5p and anti-miR-17-5p detected by qRT-PCR. **b** The expression level of Smad7 in mice oral mucosa tissues treated after PBS or Bleomycin exposure. n=6 for each group. **c** The relationship between relative Smad7 expression normalized to GAPDH and miR-17-5p expression normalized to U6. Statistics: mean ± SEM, unpaired, two-tailed Student’s t-test (b) or one-way ANOVA (a), *p < 0.05, **p < 0.01.

**Supplementary Figure 6** miR-17-5p interferes with the function of Smad7-mediated TGFBR1 expression. **a, b** HEK293T transfected with Smad7 and then incubated with CHX for indicated time points. The protein levels of TGFBR1 were determined at indicated time points by Immunoblotting (a) and a corresponding quantification curve was exhibited (b). **c** HEK293T and fibroblast co-transfected with Smad7 and miR-17-5p. The protein levels of TGFBR1 were determined by Immunoblotting. **d, e** HEK293T (d) and fibroblasts (e) were transfected with miR-17-5p and the binding of TGFBR1 to Smad7 was determined by Co-IP and Immunoblotting.

**Supplementary Figure 7** Exogenous Co-IP and Immunoblotting detected the interaction of WWP1 and TGFBR1 in HEK293T.

**Supplementary Figure 8** miR-17-5p aggravated skin fibrosis in mice. **a** Schematic overview of mouse experimental design. Mice were injected with 50 μL PBS or Bleomycin into the back skin every other day for six weeks. Then, mice were simultaneously treated with miR-NC (Ctrl), miR-17-5p agomir and SB525334 for another two weeks. **b** HE, Masson and Sirius red staining of mice skin tissues. Scale bar, 200 μm. Quantification of Masson staining for collagen fraction. Scale bar, 200 μm. **c** Quantification of Masson staining for skin tissues collagen fraction, n=6 for each group. **d** Hydroxyproline content of mice skin tissues. **e** Immunoblotting of fibrotic markers (Fibronectin, collagen type I and α-SMA), Smad7, TGFBR1 and p-Smad2 in mice skin tissue. Statistics: mean ± SEM, n=6 for each group, one-way ANOVA, *p < 0.05, **p < 0.01, ***p < 0.001, ****p < 0.0001.

**Supplementary Table 1.** The primer sequences used for qRT-PCR.

| Gene |  | Primer Sequence (5’-3’) |
| --- | --- | --- |
| *COL1A1 (Homo)* | Forward | GAGGGCCAAGACGAAGACATC |
|  | Reverse | CAGATCACGTCATCGCACAAC |
| *ACTA2 (Homo)*  *Smad7 (Homo)*  *TGFBR1 (Homo)*  *NEDD4L (Homo)*  *SMURF1 (Homo)*  *SMURF2 (Homo)*  *TRAF6 (Homo)*  *VHL (Homo)*  *WWP1 (Homo)*  *GAPDH (Homo)*  *Smad7 (Mus)*  *GAPDH (Mus)* | Forward  Reverse  Forward  Reverse  Forward  Reverse  Forward  Reverse  Forward  Reverse  Forward  Reverse  Forward  Reverse  Forward  Reverse  Forward  Reverse  Forward  Reverse  Forward  Reverse  Forward  Reverse | GGCATTCACGAGACCACCTAC  CGACATGACGTTGTTGGCATAC  TTCCTCCGCTGAAACAGGG  CCTCCCAGTATGCCACCAC  GGCCAGATCCTGTCCAAGC  GTGGGTTTCCACCATTAGCAC  TAGCCTCAGCTCGCCAACAGTA  GGAGTTGTAAGGTGATGGCTGTG  AGTCCTCAGACACGAACTGTCG  GTCGCATCTTCATTATCTGGCGG  TCCTCGGCTGTCTGCTAACTTG  CAGGCATTCTGTGTCATCAGGAC  CAATGCCAGCGTCCCTTCCAAA  CCAAAGGACAGTTCTGGTCATGG  GACACACGATGGGCTTCTGGTT  ACAACCTGGAGGCATCGCTCTT  TGAACAGTGGCAATCTCAGCGG  CTGGTGGCAAAGGTCCATAAGG  ACAACTTTGGTATCGTGGAAGG  GCCATCACGCCACAGTTTC  TTCTCAAACCAACTGCAGGCT  CCGGCTGTTGAAGATGACCT  CATCACTGCCACCCAGAAGACTG  ATGCCAGTGAGCTTCCCGTTCAG |
