# Supplementary figures and images for: Exosomal miR-17-5p derived from epithelial cells is involved in aberrant epithelium-fibroblast crosstalk and induces the development of oral submucosal fibrosis
Source: Int J Oral Sci. 2024 Jun 20;16:48. doi: 10.1038/s41368-024-00302-2 (PMC11187069; doi:10.1038/s41368-024-00302-2)

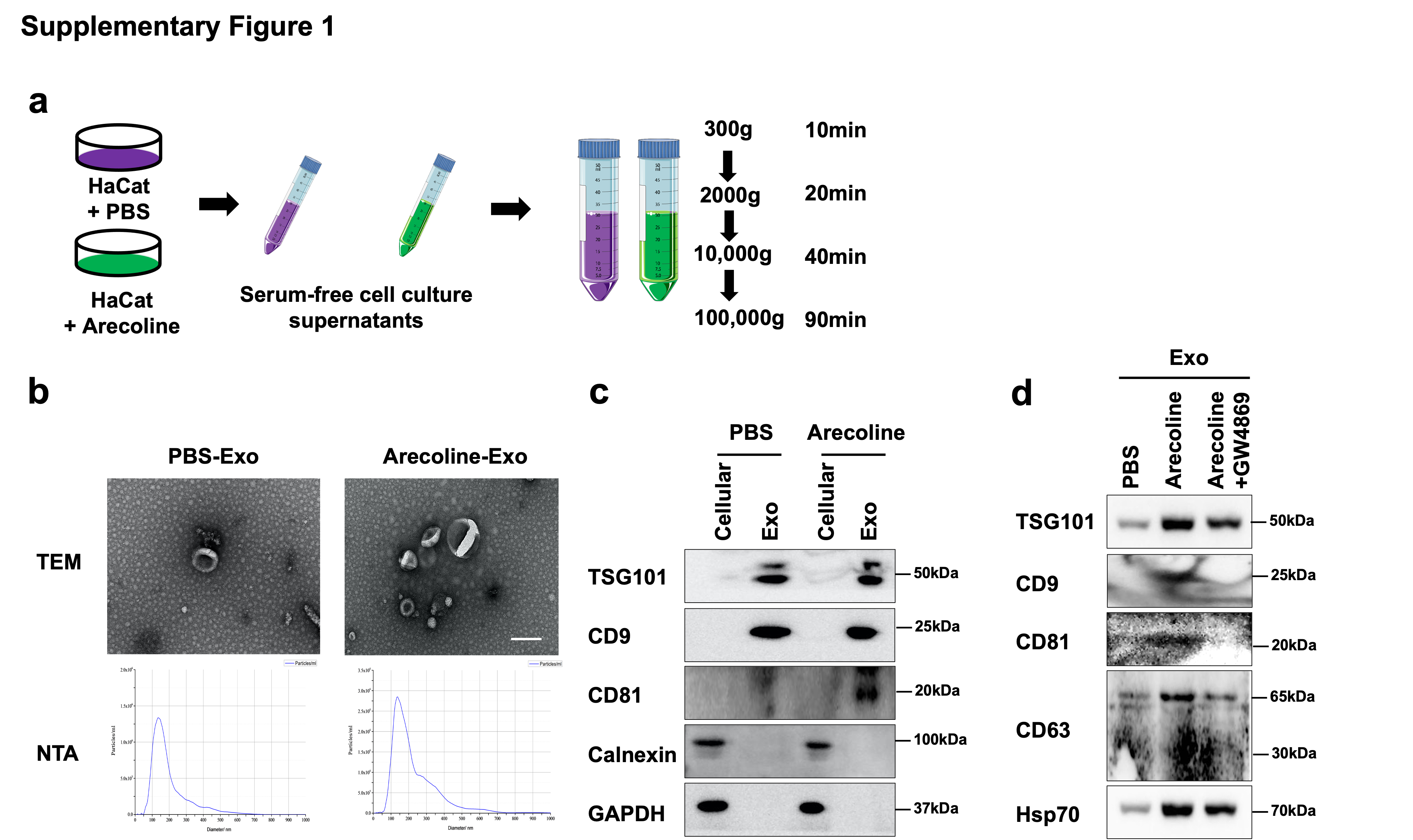

Supplement: Supplementary file 2 — Supplementary Figure 1 [file 41368_2024_302_MOESM2_ESM.jpg]

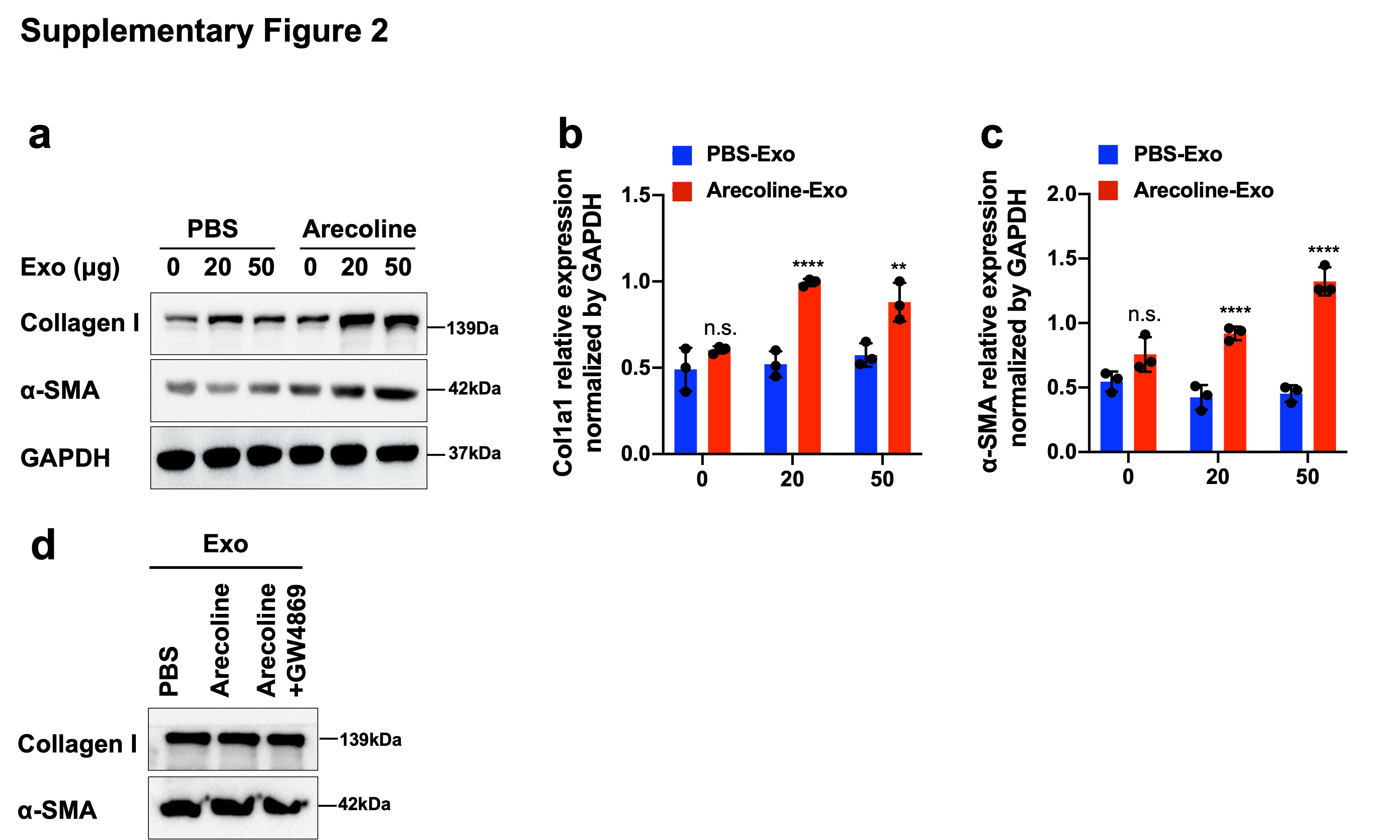

Supplement: Supplementary file 3 — Supplementary Figure 2 [file 41368_2024_302_MOESM3_ESM.jpg]

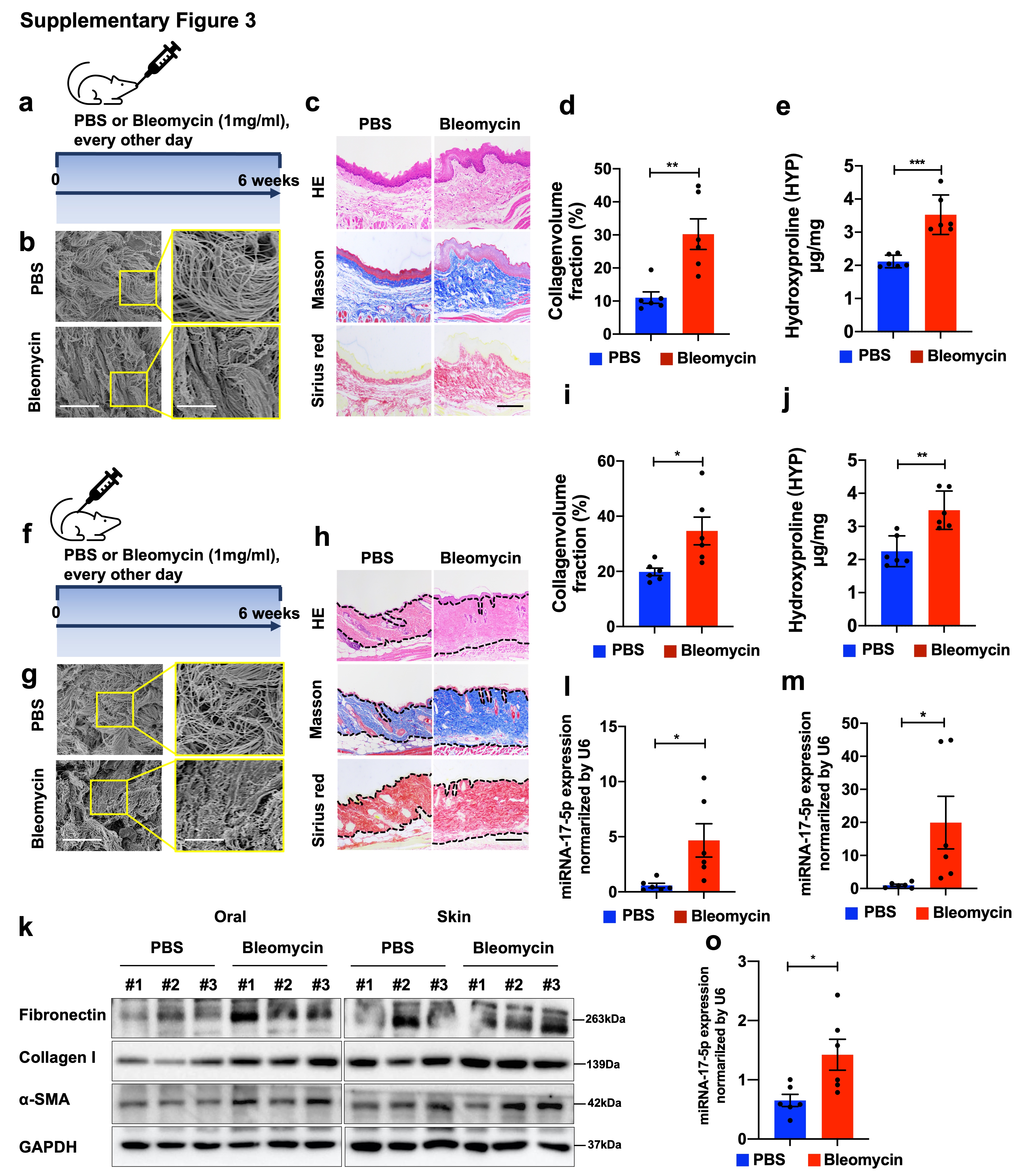

Supplement: Supplementary file 4 — Supplementary Figure 3 [file 41368_2024_302_MOESM4_ESM.jpg]

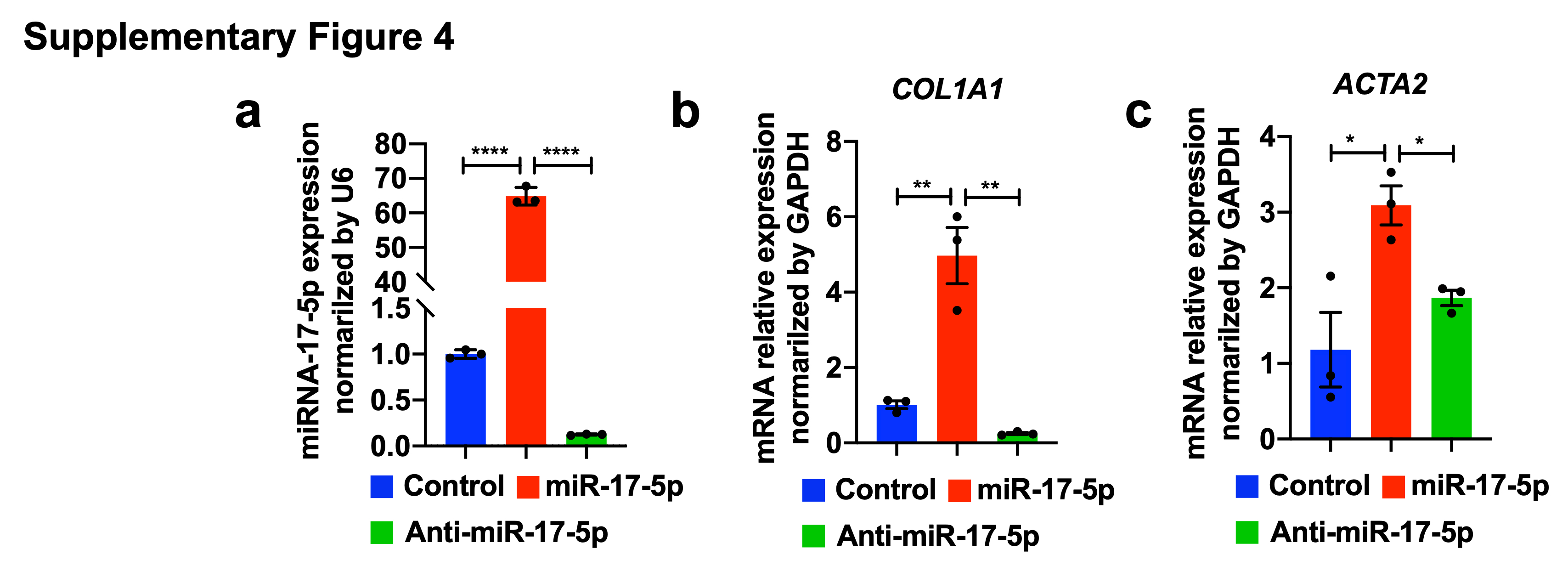

Supplement: Supplementary file 5 — Supplementary Figure 4 [file 41368_2024_302_MOESM5_ESM.jpg]

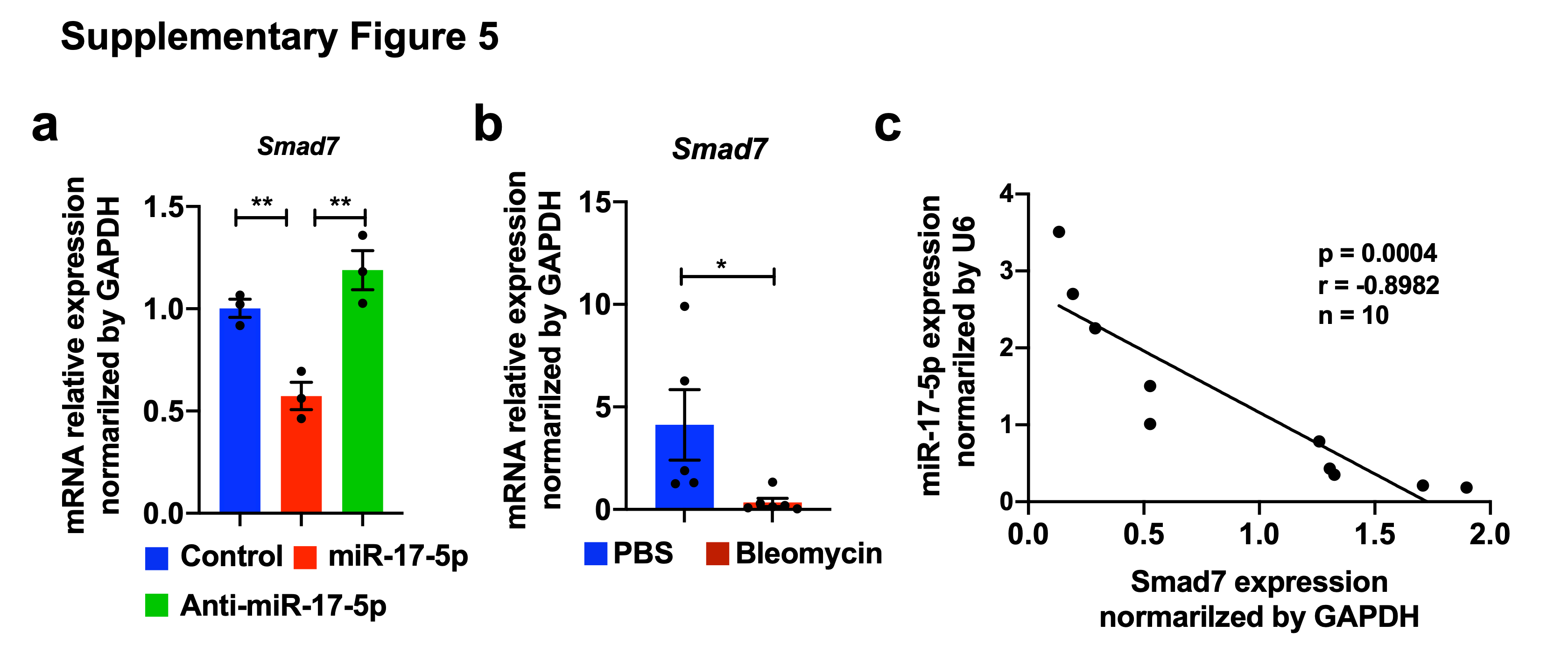

Supplement: Supplementary file 6 — Supplementary Figure 5 [file 41368_2024_302_MOESM6_ESM.jpg]

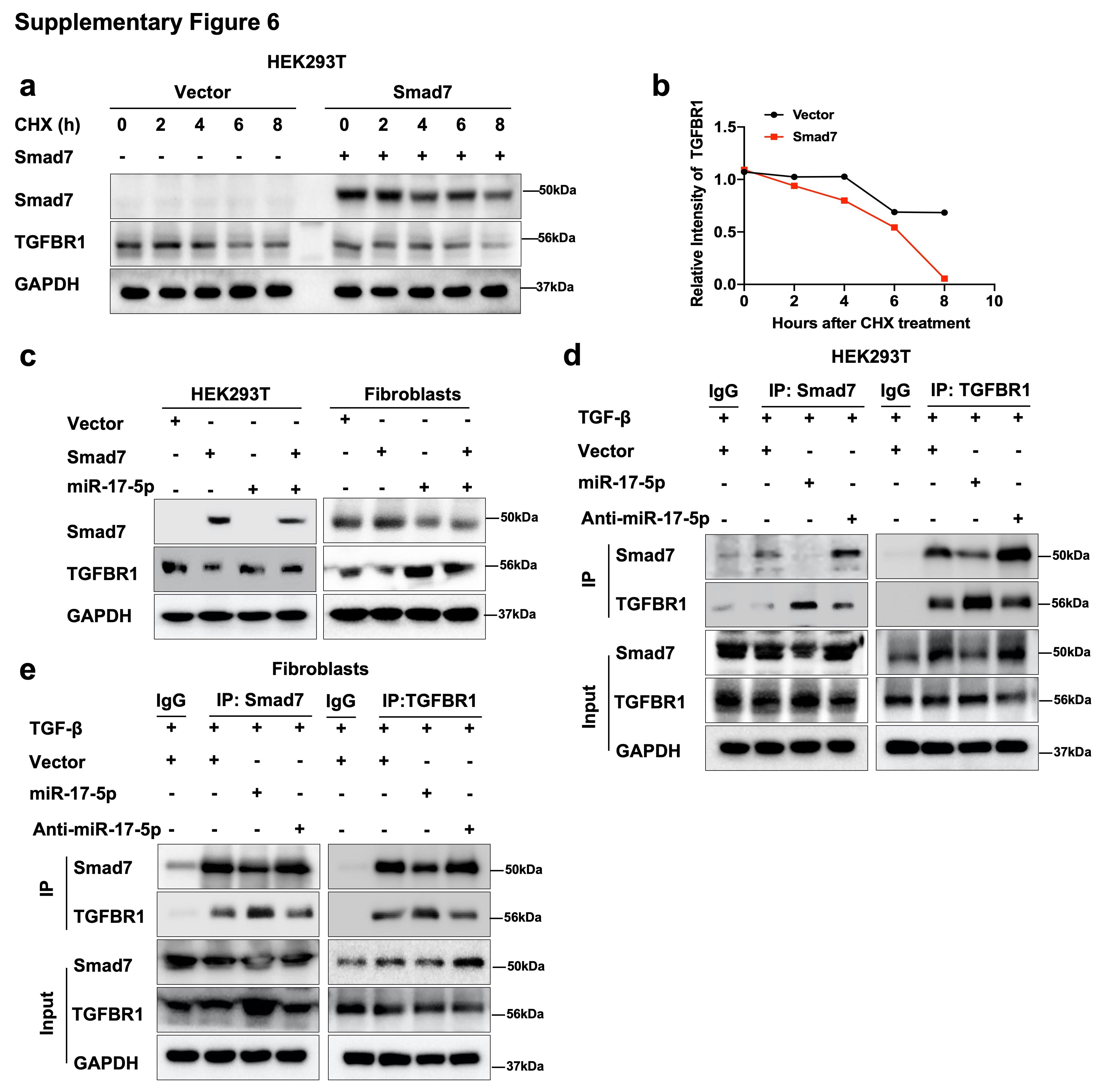

Supplement: Supplementary file 7 — Supplementary Figure 6 [file 41368_2024_302_MOESM7_ESM.jpg]

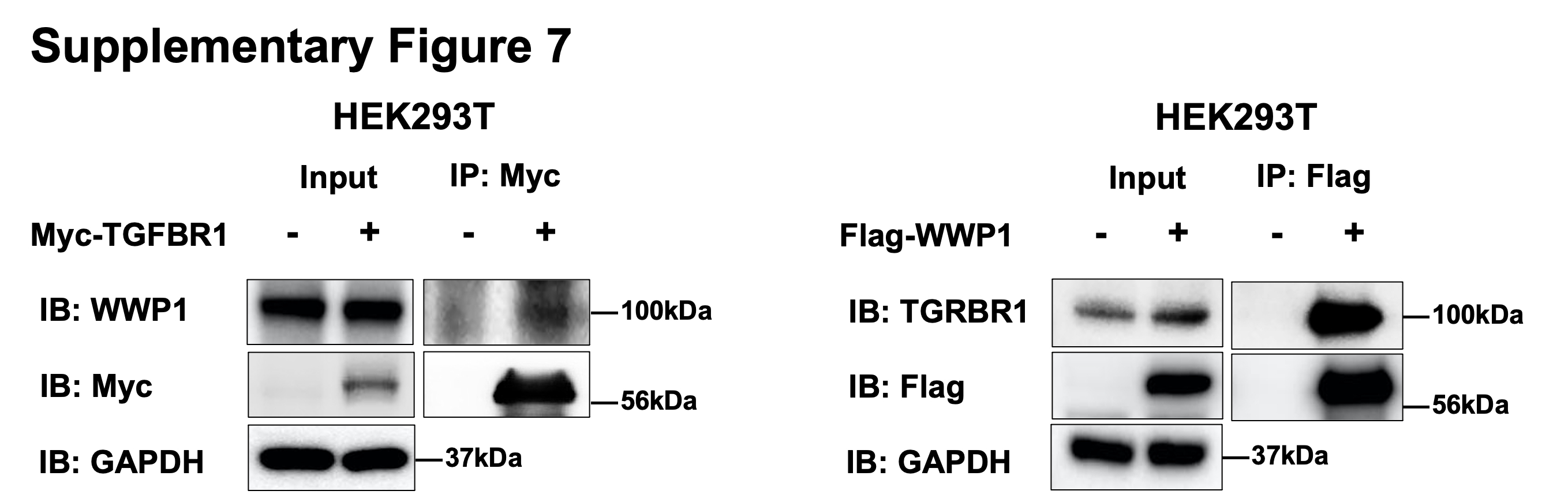

Supplement: Supplementary file 8 — Supplementary Figure 7 [file 41368_2024_302_MOESM8_ESM.jpg]

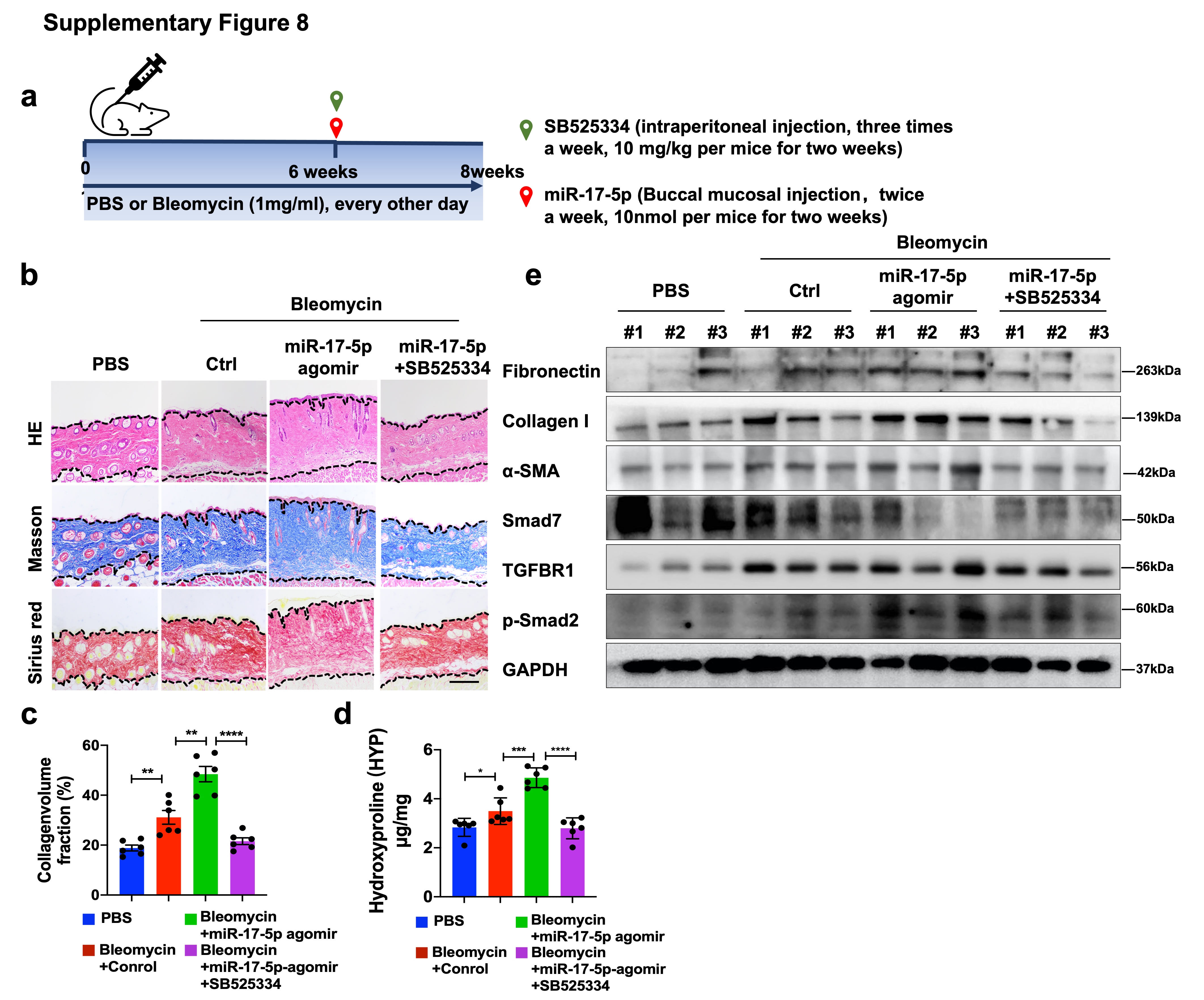

Supplement: Supplementary file 9 — Supplementary Figure 8 [file 41368_2024_302_MOESM9_ESM.jpg]
